# Using an Online Sample to Estimate the Size of an Offline Population

**DOI:** 10.1007/s13524-019-00840-z

**Published:** 2019-12-03

**Authors:** Dennis M. Feehan, Curtiss Cobb

**Affiliations:** 1 Department of Demography, University of California, Berkeley, Berkeley, CA USA; 2 Facebook, Inc., 1 Hacker Way, Menlo Park, CA 94025 USA

**Keywords:** Networks, Sampling, Digital demography, Digital divide, Survey research

## Abstract

Online data sources offer tremendous promise to demography and other social sciences, but researchers worry that the group of people who are represented in online data sets can be different from the general population. We show that by sampling and anonymously interviewing people who are online, researchers can learn about both people who are online and people who are offline. Our approach is based on the insight that people everywhere are connected through in-person social networks, such as kin, friendship, and contact networks. We illustrate how this insight can be used to derive an estimator for tracking the *digital divide* in access to the Internet, an increasingly important dimension of population inequality in the modern world. We conducted a large-scale empirical test of our approach, using an online sample to estimate Internet adoption in five countries (*n* ≈ 15,000). Our test embedded a randomized experiment whose results can help design future studies. Our approach could be adapted to many other settings, offering one way to overcome some of the major challenges facing demographers in the information age.

**Electronic supplementary material:**

The online version of this article (10.1007/s13524-019-00840-z) contains supplementary material, which is available to authorized users.

## Introduction

Online data sources offer tremendous promise to demography and other social sciences (Cesare et al. 2018; Lazer et al. 2009; Zagheni and Weber 2012), but researchers often worry that the group of people who are represented in online data sets can be different from the general population. In this study, we develop a strategy for addressing this challenge: we show that by sampling and anonymously interviewing people who are online, researchers can learn about both people who are online and people who are offline.

Asking survey respondents to report about others is an idea that has independently arisen in many substantive areas (see, e.g., Bernard et al. 1991; Hill and Trussell 1977; Marsden 2005; Sirken 1970). In demography, the approach can be traced back to Brass’s innovative development of census and survey questions that ask respondents about their parents, spouses, or siblings (Brass 1975). Our approach can be seen as an extension of this previous work to research in which the goal is to learn about everyone in a population but respondents are sampled and interviewed only online. Thus, our study is an illustration of one way to overcome many challenges that face the sampling and survey research community in the information age.

We illustrate our methodology by developing a new way to study the digital divide in access to the Internet around the world. Scholars use the term *digital divide* to refer to the fact that access to the Internet is highly unequal: billions of people around the world have never been online (Hjort and Poulsen 2019; World Bank 2016); people in poor countries use the Internet much less than people in wealthy countries (World Bank 2016); and even within countries that enjoy high levels of Internet adoption, research suggests that access to the Internet can differ considerably by age, gender, income, and race (Friemel 2016; Haight et al. 2014; Van Deursen and Van Dijk 2014; Vigdor et al. 2014). Thus, the digital divide is an important dimension of population inequality in the modern world.

The digital divide is important because research has revealed that access to the Internet may affect health and well-being through a wide range of different mechanisms. For example, scholars have found that increasing Internet adoption may lead to job creation (Hjort and Poulsen 2019), improvements in education (Kho et al. 2018), increases in international trade (Clarke and Wallsten 2006), increases in social capital (Bauernschuster et al. 2014), political mobilization (Manacorda and Tesei 2016), reduced sleep (Billari et al. 2018), and changes in fertility (Billari et al. 2019). The World Bank devoted its 2016 World Development Report to the digital dividends that may result from increasing access to the Internet in the developing world (World Bank 2016).

Reliable estimates of Internet adoption are typically based on methodologically rigorous household surveys or censuses (e.g., Cohen and Adams 2011; ICF 2004). However, this rigor comes at a price: these surveys can be very costly and typically take months to design and implement (e.g., Greenwell and Salentine 2018; ICF 2018; Parsons et al. 2014; Rojas 2015). These limitations are especially problematic because Internet adoption appears to be changing on a much faster time scale than many conventional indicators of social and economic well-being (Perrin and Duggan 2015; World Bank 2016).

The difficulty of obtaining up-to-date estimates of Internet adoption is unfortunate because researchers need to be able to measure the digital divide to understand its implications for inequality and opportunity; and policymakers who want to implement and evaluate strategies for making Internet access more widely available rely on being able to measure the level and rate of change in the number of people who have access to the Internet.^1^

To help address this challenge, we used our methodology to develop an alternative approach to estimating Internet adoption that is dramatically faster and less expensive than conventional surveys: we interviewed a sample of Facebook users and asked them whether members of their offline personal networks use the Internet. Our approach is based on the insight that Internet users are connected to many other people through in-person social networks such as kin, friendship, and contact networks. By interviewing a sample of Facebook users and anonymously asking about the members of these offline social networks, we can learn about both people who are online and people who are not.

## Methods

People everywhere are connected to one another through kinship, friendship, professional activities, and interpersonal interactions. Our strategy for obtaining fast and inexpensive estimates of Internet adoption is based on asking people sampled online to report about Internet adoption among other people they are connected to in these everyday, offline personal networks. The challenge is to determine how to turn people’s anonymous reports about their personal network members into estimates of Internet adoption. We used a formal framework called *network reporting* to understand which quantities we need to estimate to accomplish our goal (Feehan 2015; Feehan and Salganik 2016a). (A detailed derivation can be found in section A of the online appendix.)

**Fig. 1:**
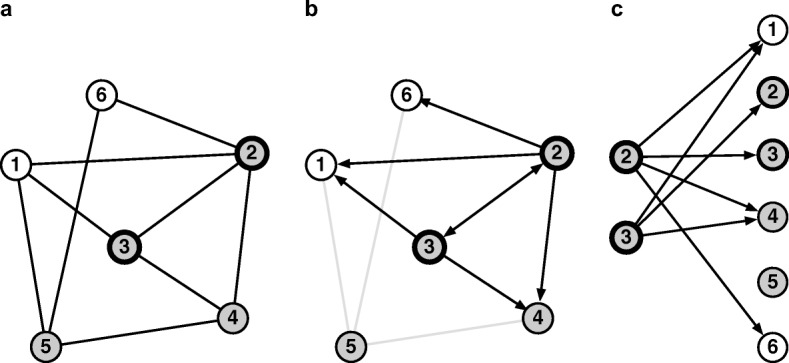
Network reporting setup: asking people on Facebook to report about their offline personal networks

Figure 1 illustrates the general setup with an example. Panel a of Fig. 1 shows six people connected in a social network. The network relation is *symmetric*, meaning that whenever person A is connected to person B, person B is also connected to person A. We distinguish between nodes that can potentially be sampled and interviewed—the *frame population*—and other nodes. For example, a frame population might be cell phone users; the users of a specific app, such as Facebook; or people who live at addresses that can be reached by postal mail. In Fig. 1, nodes 2 and 3 are in the frame population.

Panel b of Fig. 1 shows the *reporting network* that is generated when both nodes 2 and 3 are interviewed about the people they are connected to in the social network. The reporting network is different from the social network: the social network has an undirected edge *A* – *B* when A and B are socially connected; the reporting network, on the other hand, has a directed edge *A* → *B* whenever A reports about B. When reporting is accurate, the social network and the reporting network will have structural similarities, but this need not be true in general. The reporting network is a useful formalism that can help researchers develop estimators, understand possible sources of reporting errors, and derive self-consistency checks.

Panel c of Fig. 1 shows a rearrangement of panel b that is helpful for deriving estimators from a reporting network. On the left side of panel c is the set of nodes that makes reports (the frame population), and on the right side is the set of nodes that can be reported about (the universe).^2^ Drawn this way, every report must connect a node on the left side to a node on the right side. Thus, the total number of reports that leaves the left side must equal the total number of reports that arrives at the right side. Mathematically, this means that when everyone in the frame population is interviewed, we have the following identity:(1)

The denominator of Eq. (1) is a quantity called the *visibility* of Internet users, which is the number of times that the average Internet user would be reported in a census of the frame population. Intuitively, Eq. (1) divides by the visibility to adjust for the fact that the average Internet user would be reported multiple times in a census of the frame population.

## Instrument Design

In principle, people can be asked to report about any type of personal network relationship that is symmetric. Thus, the specific type of personal network that respondents are asked to report about—the *tie definition*—is a study design parameter that researchers are free to vary (Feehan et al. 2016). To explore the impact of this study design parameter, we embedded a randomized experiment in our survey. In our experiment, survey respondents were randomly assigned to report about one of two tie definitions: the meal tie definition and the conversational contact tie definition (Table 1). We chose these two tie definitions for two reasons. First, previous research led us to believe that respondents can plausibly report the number of people that they interacted with in the previous day, avoiding the need to indirectly estimate personal network sizes. Second, researchers have had success using versions of these tie definitions in previous studies (Feehan et al. 2016; Mossong et al. 2008).

**Table 1:** The two networks about which respondents were surveyed^a^

Meal Network	Conversational Contact Network
How many people did you share food or drink with yesterday? These people could be family members, neighbors, or other people. Please include all food or drink taken at any location, including at home, at work, at a cafe, or in a restaurant.	How many people did you have conversational contact with yesterday? By conversational contact, we mean anyone you spoke with face to face for at least three words.

^a^ In our survey experiment, respondents were randomly assigned to report about one of these two networks.

Each survey interview took place in two phases. In the first phase, survey respondents were asked to report the size of their personal networks: for example, “How many people did you share food or drink with yesterday?” (Table 1). In the second phase, the goal was to obtain information about Internet use among the members of each respondent’s personal network. Ideally, the respondent would provide information about every person in her network one by one. However, this approach seemed likely to produce unacceptable levels of respondent fatigue (Eckman et al. 2014; Tourangeau et al. 2015). Therefore, in the second phase of the interview, respondents were asked for information about the three members of their personal networks who came to mind first (Fig. S6, online appendix). We call these people for whom we obtain additional information *detailed alters*.^3^ Additional details and our survey instrument are included in section D of the online appendix.

## Estimators

The identity in Eq. (1) would hold if we had obtained a census of monthly active Facebook users. In practice, we have a sample and not a census; therefore, we construct an estimator for the number of Internet users by developing sample-based estimators for the numerator and the denominator of Eq. (1). We now describe these two components in more detail.

Given information about respondents’ network sizes and the detailed alters’ Internet use, the numerator of 1 (*y*_*F*,*H*_) can be estimated from our sample with

2 $$ {\hat{y}}_{F,H}={\sum}_{i\in s}{w}_i\frac{d_i}{r_i}{o}_i, $$

where *s* is the sample of Facebook users; *w*_*i*_ is the expansion weight for *i* ∈ *s*; *d*_*i*_ is the network size (degree) of *i* ∈ *s*; *r*_*i*_ is the number of detailed alters from *i* ∈ *s* (*r*_*i*_ ∈{1*,* 2*,* 3}); and *o*_*i*_ is the number of detailed alters reported to be online.

We calculate *w*_*i*_ by approximating our design as a simple random sample, post-stratified by age and gender. (Section D of the online appendix has more information on our weighting.) To use information about the *r*_*i*_ detailed alters to make inferences about the *d*_*i*_ people in the respondent’s network, the estimator in Eq. (2) makes the additional assumption that the detailed alters are a simple random sample of respondents’ personal networks. Thus, *d*_*i*_ / *r*_*i*_ can be seen as a weight that accounts for sampling *r*_*i*_ of the *d*_*i*_ personal network members. Previous work on egocentric survey research suggests that instead of being a simple random sample, network members who come to mind first may be more likely to come from the same social context and may be more likely to be strongly connected to the respondent (Marsden 2005). Therefore, we develop two ways to assess this assumption. First, we introduce internal consistency checks that can detect systematic biases that would emerge if detailed alters are very different from other personal network members. Second, we introduce a sensitivity framework that enables us to formally assess the impact that different magnitudes of selection bias among the detailed alters would have on our estimates (online appendix, section C).

The denominator of Eq. (1) (${\overline{v}}_{H,F}$) is a quantity called the *visibility* of Internet users, which is defined as the number of times that the average Internet user would be reported in a census of active Facebook users. Many different strategies could be used to estimate or approximate the visibility of Internet users. Here, we adopt a simple approach: we use the average number of times that a Facebook user shares a meal with another Facebook user to approximate the visibility of Internet users. Mathematically, this assumption can be written

3 $$ {\overline{d}}_{H,F}={\overline{d}}_{F,F}. $$

The condition in Eq. (3) requires that two quantities be equal: (1) the rate at which someone who is on the Internet shares a meal with someone who is on Facebook (${\overline{d}}_{H,F}$) and (2) the rate at which someone who is on Facebook shares a meal with someone who is also on Facebook (${\overline{d}}_{F,F}$). This assumption would hold if, for example, people who are on the Internet do not pay attention to whether another Internet user is on Facebook when deciding to share a meal. This assumption could be violated if, for example, people frequently organize sharing a meal using Facebook without inviting other people. We explore how violating this condition affects estimates as part of a sensitivity analysis in section C of the online appendix; in section F of the online appendix, we develop a simple model that motivates this condition; and in the Conclusion, we discuss how additional data collection could remove the need for this condition altogether.

Given the condition in Eq. (3), we can estimate ${\overline{v}}_{H,F}$ with an estimator for ${\overline{d}}_{F,F}$, the average number of meals that someone on Facebook reports sharing with someone else on Facebook. To estimate ${\overline{d}}_{F,F}$, we use

4 $$ {\hat{\overline{d}}}_{F,F}=\frac{\sum_{i\in s}{w}_i\frac{d_i}{r_i}{f}_i}{\sum_{i\in s}{w}_i}, $$

where the new quantity, *f*_*i*_, is the number of Facebook users that respondent *i* reports among her detailed alters.

Putting Eq. (2) and Eq. (4) together, we have

5 $$ {\hat{N}}_H=\frac{{\hat{y}}_{F,H}}{{\hat{\overline{d}}}_{F,F}}=\frac{\sum_{i\in s}{w}_i\frac{d_i}{r_i}{o}_i}{\sum_{i\in s}{w}_i\frac{d_i}{r_i}{f}_i}\times {\sum}_{i\in s}{w}_i. $$

Section A of the online appendix has a detailed derivation of the estimator and a precise description of all the conditions on which it relies; section E describes an alternate approach to producing estimates using data we collected; and section C has a framework for sensitivity analysis that can be used to understand how estimates are affected by violations of these conditions.

## Results

We used Facebook’s survey infrastructure to obtain a simple random sample of people who actively use Facebook in five countries around the world: Brazil (*n* = 3,761), Colombia (*n* = 4,157), Great Britain (*n* = 781), Indonesia (*n* = 2,794), and the United States (*n* = 4,288).^4^ We chose these countries because they span a breadth of expected levels of Internet adoption and economic development. The sample contains slightly more female than male respondents in all countries except for Indonesia, and age distributions are typical of monthly active Facebook users in these countries.

**Fig. 2:**
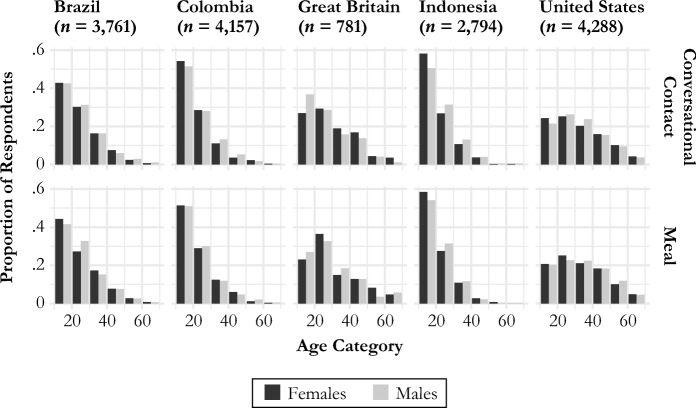
Age and gender of survey respondents in each country. Estimates throughout this article use sampling weights to account for sample design and nonresponse.

Figure 2 shows the age and gender distribution of survey respondents for each tie definition.^5^ All estimates are weighted to account for the sample design and to be representative of the universe of monthly active Facebook users in each country. Estimates of sampling uncertainty are based on the rescaled bootstrap method (Feehan and Salganik 2016b; Rao and Wu 1988; Rao et al. 1992).

**Fig. 3:**
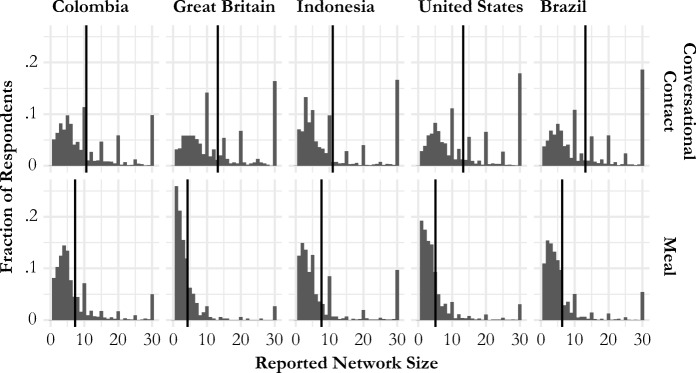
Estimated degree distributions for the conversational contact network (top panels) and the meal network (bottom panels). The vertical line on each panel shows the average. Average personal network size is smaller for the meal network than for the contact network; further, the contact network shows greater evidence of heaping on multiples of 5 and 10 than the meal network. These findings are consistent with a hypothesized trade-off between the quality and the quantity of information reported in personal networks. Responses higher than 30 are coded as 30 in these plots.

Figure 3 shows the distribution of personal network sizes reported by respondents from each country and for each tie definition.^6^ The average size of meal networks was smaller than conversational contact networks in all countries (Table S2, online appendix). The average reported size of the meal network varied from about 4 (Great Britain) to about 8 (Indonesia). The average reported size of the conversational contact network varied from about 11 (Colombia and Indonesia) to about 13 (Brazil, Great Britain, and the United States). For both networks, Fig. 3 suggests that there may be heaping in reported network sizes that are multiples of 5 and 10; this heaping is more evident in the reported number of conversational contacts than for meals, suggesting that reports about the meal network may be more accurate.

## Internal Consistency Checks

To more formally assess the accuracy of reports about each network, we developed *internal consistency checks* (Bernard et al. 2010; Brewer et al. 2000; Feehan et al. 2016) using the information about the age group and gender of each detailed alter from respondents’ reports. The idea is to find reported quantities that can be estimated from the data in two ways. To the extent that these independent estimates of the same quantity agree, the reported network connections are internally consistent. For example, using survey responses from only men, we could estimate the number of connections between men and women; similarly, using survey responses from only women, we could estimate the number of connections between women and men. By definition, these two quantities are equal; thus, under perfect conditions in which our survey does not suffer from any reporting errors or selection biases, we would expect these two independent estimates to agree (up to sampling noise).

We devised internal consistency checks based on reported connections to and from each of six age-sex groups, by country and by tie definition. For each age-sex group α, we estimated the average number of connections from Facebook users in age-sex group α to Facebook users not in $\upalpha \left({d}_{F_{\upalpha},{F}_{-\upalpha}}\right)$. We also estimated the average number of connections from Facebook users not in age-sex group α to Facebook users who are in age-sex group $\upalpha \left({d}_{F_{-\upalpha},{F}_{\upalpha}}\right)$. We then defined the average normalized difference *∆*_α_ to be

6 $$ {\Delta }_{\upalpha}=K\left({\hat{d}}_{F_{-\upalpha},{F}_{\upalpha}}-{\hat{d}}_{F_{\upalpha},{F}_{\hbox{--} \upalpha}}\right), $$

where *K* is a scaling factor intended to ease comparison of different countries and age-sex groups (online appendix, section B). In the absence of any reporting error, selection biases, or sampling variation, we would expect *∆*_α_ = 0. On the other hand, if there is homophilic selection bias in the respondents’ choice of detailed alters or if members of group α are especially conspicuous, then we would expect *∆*_α_*>* 0. Similarly, if there is heterophilic selection bias in respondents’ choice of detailed alters or if members of a group are especially inconspicuous, then we would expect *∆*_α_ *<* 0.

**Fig. 4:**
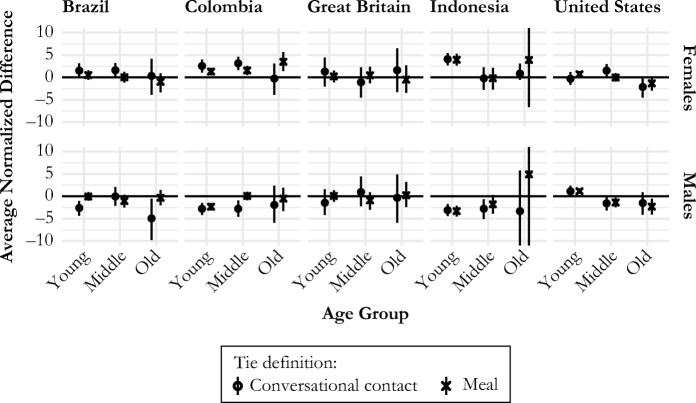
Internal consistency checks. By estimating the same quantity using independent parts of our sample, we can assess the internal consistency of respondents’ network reports. Estimated difference between two independent estimates of the same quantity and 95% confidence intervals are shown for each age-sex group and each type of network; an estimate of 0 means that the two independent estimates are exactly the same. Across most age-sex groups, results are internally consistent, within sampling error; however, some groups show evidence of reporting errors (e.g., young people in Indonesia). Results also suggest that reports about the meal definition are more internally consistent, even though meal networks are smaller than conversational contact networks.

Figure 4 shows the average normalized difference (*∆*_α_) for internal consistency checks based on reported connections to and from each of six age-sex groups, by country and by tie definition. Several notable features emerge from Fig. 4. First, for many of the internal consistency checks, the averaged normalized differences are close to 0 or have confidence intervals that contain 0. Second, Fig. 4 suggests that reports based on the meal network are, on average, more internally consistent than reports based on conversational contact (confirmed in section G of the online appendix). Third, there appears to be no universal pattern that describes deviations in internal consistency checks that are not close to 0. Taking the example of Indonesia, the average normalized differences for younger age groups suggest that young women may be relatively conspicuous or that young women are relatively homophilous.^7^ On the other hand, young men are relatively inconspicuous or relatively heterophilous. In Brazil and Colombia, similar patterns appear for the conversational contact network. In Great Britain and the United States, however, most of the internal consistency checks suggest that reports are internally consistent.

## Comparing Tie Definition Accuracy

**Fig. 5:**
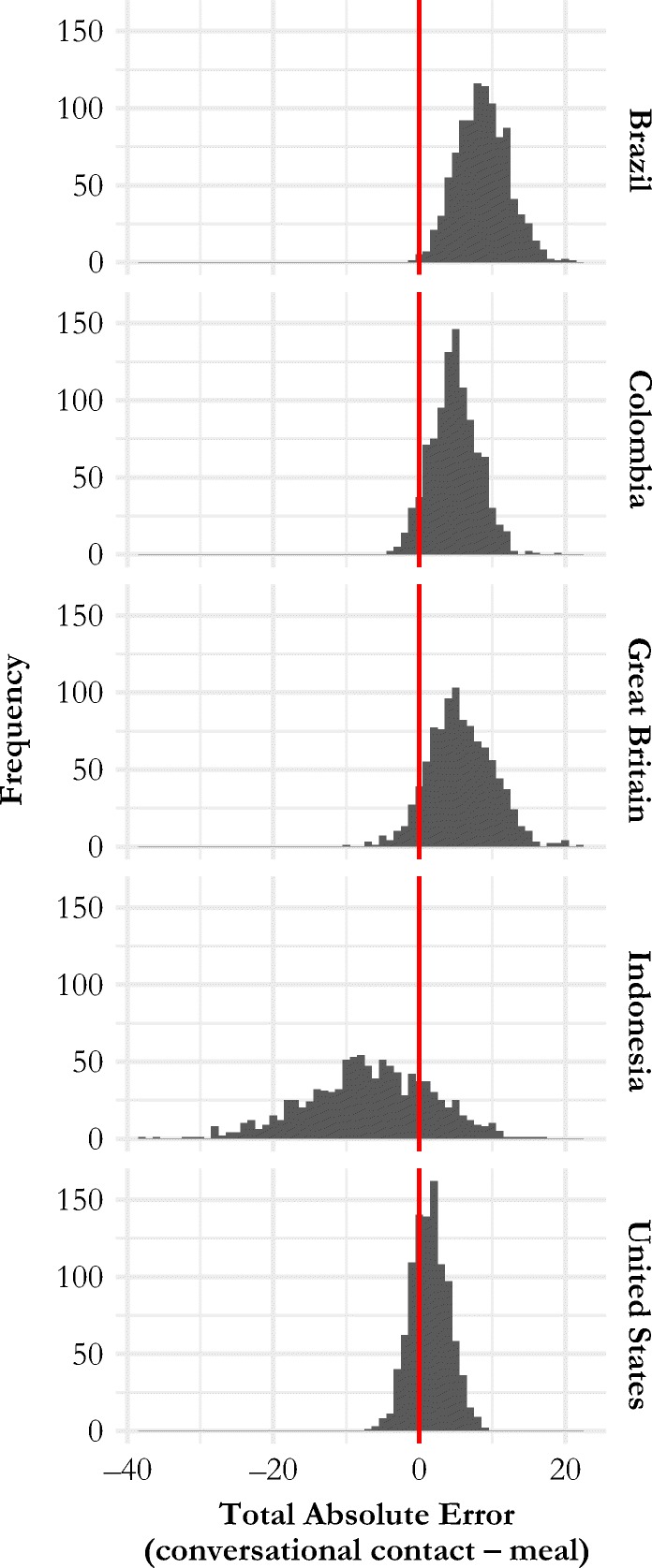
Estimated sampling distribution of the difference between the total absolute error (TAE) for internal consistency checks from the conversational contact network and from the meal network. For all countries except for Indonesia, the meal network is more internally consistent than the conversational contact network (Table S3, online appendix).

Figure 5 directly compares the difference in internal consistency results for the conversational contact and meal networks. The figure shows the estimated sampling distribution of TAE, the total absolute error difference between the internal consistency checks for the conversational contact network and the internal consistency checks for the meal network:

7 $$ \mathrm{TAE}={\sum}_{\upalpha}\left(\left|{\Delta }_{\upalpha, \mathrm{cc}}\right|-\left|{\Delta }_{\upalpha, \mathrm{meal}}\right|\right), $$

where |*∆*_α, cc_| and |*∆*_α, meal_| are the absolute internal consistency check statistics based on group α for the conversational contact and meal networks (i.e., the absolute value of Eq. (6)). Thus, TAE is a summary of how well the internal consistency checks perform across all age-sex groups for the conversational contact network minus the meal network. Because values of |*∆*_α_| close to 0 indicate more internally consistent reports, a positive TAE suggests that the meal network is more internally consistent; conversely, a negative TAE suggests that the conversational contact network is more internally consistent. For all countries except for Indonesia, the majority of the mass of the estimated distribution is greater than 0, suggesting that the meal network reports are more internally consistent than conversational contact network reports (Table S3).

## Estimates of Internet Adoption

**Fig. 6:**
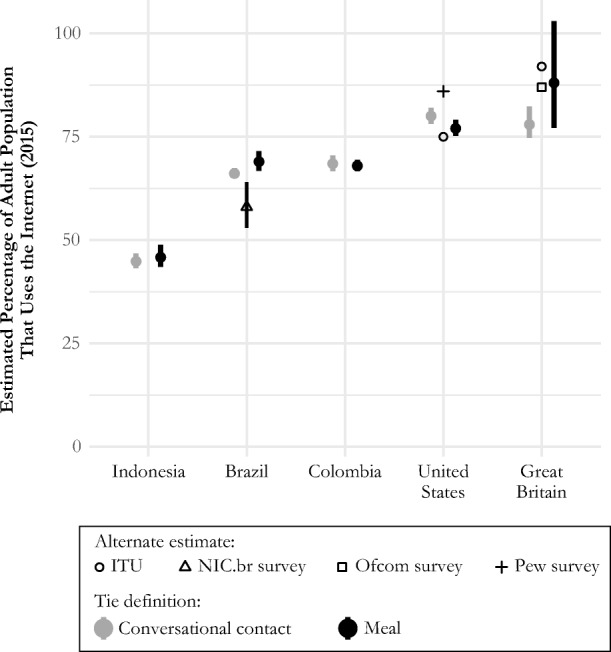
Estimated percentage of 2015 adult population that uses the Internet, by country and for each of the two networks. The 95% confidence intervals are based on the estimated sampling distribution from the rescaled bootstrap. For comparison, estimates from alternate sources are shown where available. In Great Britain, comparison estimates are available from the ITU and an Ofcom survey; in the United States, comparison estimates are available from the ITU and Pew Research Center surveys; and in Brazil, comparison estimates are available from an NIC.br survey. The confidence interval around the Brazil estimate is based on the survey’s published margin of error.

Figure 6 shows estimated Internet adoption for each country in our sample, using each tie definition.^8^ Two findings emerge from Fig. 6. First, estimated Internet adoption rates are very similar for the conversational contact and for the meal networks; in all countries, the confidence intervals for estimates from the two tie definitions overlap. Second, the countries can be divided into three groups according to estimated adoption rates: the United States and Great Britain have the highest rates of Internet adoption (above 75%); Brazil and Colombia have estimated Internet adoption rates between 50% and 75%; and Indonesia has estimated adoption rates below 50%. This ordering is consistent with what would be predicted if economic factors such as GDP per capita were the main driver of Internet adoption.

Ideally, we would evaluate our estimator by comparing it with gold standard measurements of Internet adoption in each of the five countries. Unfortunately, no such gold standard exists. Therefore, to further assess the plausibility of the estimates presented in Fig. 6, we compared our results with existing Internet adoption estimates for Great Britain, the United States, and Brazil, the countries where high-quality alternative estimates were available.^9^ The results show that the fast and inexpensive network reporting estimates are within the range of other estimates in the United States, similar to or slightly lower than other estimates in Great Britain, and somewhat higher than the other estimate for Brazil.

## Summary and Discussion

We found that estimates of Internet adoption from the two different networks were very similar (Fig. 6). We could not validate our estimates by comparing them with gold-standard measurements of Internet adoption rates because such a gold standard was not available. However, a comparison with high-quality alternative estimates in the United States, Great Britain, and Brazil showed that the network reporting estimates are consistent with other sources of estimates in the United States, slightly higher than the other estimate for Brazil, and consistent or slightly lower than other estimates from Great Britain (Fig. 6). Thus, we conclude that our fast and inexpensive strategy for obtaining approximate estimates of Internet adoption is promising.

We also found that in all five countries, reports from the stronger network tie (meals) produced information about fewer people than the weaker network tie (conversational contact). However, reports from the stronger network tie produced, on average, more accurate information than reports from the weaker tie in all countries except for Indonesia (Fig. 5). These findings are consistent with a hypothesized trade-off between the quantity and quality of information produced by network reports; previous work found support for this theory in network reports about interactions in the 12 months before the interview (Feehan et al. 2016). We found that this tie strength trade-off may operate even when reports are about interactions that took place the day before the interview. Future research could compare different time windows to see whether the hypothesized trade-off between the quantity and quality of information operates across time within a fixed type of network tie. We hope that a deeper understanding of the relationship between reporting accuracy and the different dimensions of network tie definitions will accumulate over time, leading to useful guidance about how to design studies like ours.

The internal consistency checks suggest that people’s reports about their network members can suffer from reporting errors and that these reporting errors vary by the individual being reported (Fig. 4). One possible mechanism for this result could be differential salience of interactions; another possible mechanism could be homophilic selection of the detailed alters. This phenomenon is important to understand for measurement and scientifically interesting in its own right; future research could explore different study designs to try to distinguish between the salience of different demographic groups on the one hand and selection bias among the detailed alters on the other. More generally, the internal consistency checks provide a way to evaluate the quality of reporting from different survey designs, enabling researchers to experiment with new designs each time data are collected. Over time, this process may help discover tie definitions that minimize reporting error (Feehan et al. 2016).

## Conclusion

We showed that a sample of people who are online can be used to estimate characteristics of a population that is not entirely online. Our approach is based on the idea that people who are sampled online can be asked to provide anonymous reports about other people to whom they are connected through different kinds of personal networks. We illustrated our approach by estimating Internet adoption in five countries. Our study included a survey experiment that can help inform future efforts to use online samples to estimate population characteristics.

Our results suggest several possible avenues for future work. In this study, we focused on simple design-based estimators. A natural next step would be to start to build more complex models using these data. These models could exploit the relationships that are embedded in the internal consistency checks as a kind of constraint, estimating adjustments to ensure that reports are internally consistent. Such a model could potentially improve the accuracy of the resulting estimates. Another next step would be to use our approach to produce estimates of Internet adoption by age and gender. Finally, future work could explore the possibility of an even simpler estimator based on asking each respondent about aggregate connections to people who use the Internet (e.g., “How many of your network members use the internet?”; Bernard et al. 2010). This approach would forgo the ability to conduct internal consistency checks and to produce estimates by age and gender, but it would be even simpler and shorter than the approach we used here.

We view our method as a complement to other promising approaches to producing population-level estimates using online samples. For example, one stream of research has focused on using changes over time among members of the online sample to estimate population changes; this approach can be useful for studying topics such as migration (e.g., Zagheni and Weber 2012). A second stream of research has used models that relate people in the online sample to the general population using covariate information observed in both sources (e.g., Fatehkia et al. 2018; Goel et al. 2015). We expect that sampling and interviewing people about members of their offline networks will be especially promising in situations where few or no people in the group being studied can be expected to be in the online sample, but we also expect that there will be situations in which these alternatives are more appropriate than network reporting. As the field of digital demography emerges, it will be important to deepen our understanding of the trade-offs between these approaches and to continue to develop new methods for producing population estimates from an online sample.

We also see our approach as a complement rather than a replacement for conventional surveys. The ideal situation would combine frequent inexpensive estimates, such as the ones described here, with less frequent conventional surveys. For example, a conventional probability sample of the general population in a country could be used to empirically estimate the average number of meals shared between an Internet user and a Facebook user; with direct estimates of that quantity, the need for a key assumption in our estimator could be completely removed. More generally, a conventional probability sample survey can be used both to assess the accuracy of the fast and inexpensive estimates and to try to measure and relax some of the assumptions required by the faster, less expensive strategy.

## Electronic Supplementary Material

ESM 1 (PDF 994 kb)

## Footnotes

1 For example, the proportion of people using the Internet in each country is one of the key indicators for the United Nations Sustainable Development Goals; see SDG indicator 17.8.1 (https://www.sdgdata.gov.au/goals/partnerships-for-the-goals/17.8.1).

2 A particular node can appear in both sides if it is in the frame population and in the universe.

3 We did not ask for any sensitive or personally identifying information about these three detailed alters.

4 We considered users to be active if they have logged onto Facebook in the 30 days before the survey; we also restricted responses to people over 15 years old.

5 To ensure that the survey instrument and methods worked well, we started with a smaller sample in Great Britain (which is why there are fewer respondents in that country).

6 Recall that respondents were randomly assigned to report either about meal networks or about conversational contact networks; thus, Fig. 3 as well as Figs. 2, 4, 5, and 6 show results broken down by tie definition.

7 Conspicuousness and homophilic reporting are not distinguishable from the data. In this discussion, we focus on conspicuousness; however, instead of Indonesian women being conspicuous, it could also be the case that Indonesian women have homophilic selection biases in choosing their detailed alters (i.e., they tend to report other women at a higher rate than would be expected from simple random sampling of their network members).

8 For this study, we say that a person has *adopted the Internet* if she used the Internet on a computer or a cell phone in the last 30 days; section D of the online appendix shows our survey instrument.

9 Our comparisons come from a Pew Research Center report (Pew Research Center 2018), which is based on a national phone survey in the United States; an Ofcom Survey in the United Kingdom (Ofcom 2016); estimates reported by the International Telecommunications Union (ITU 2018); and a household survey conducted by NIC.br in Brazil (NIC.br. 2016). The ITU estimate for the United States has all people over age 3 in the denominator, and the NIC estimate for Brazil has all people over age 10 in the denominator. All other estimates are for adults.

## References

[CR1] Bauernschuster S, Falck O, Woessmann L (2014). Surfing alone? The internet and social capital: Evidence from an unforeseeable technological mistake. Journal of Public Economics.

[CR2] Bernard H. R., Hallett T., Iovita A., Johnsen E. C., Lyerla R., McCarty C., Mahy M., Salganik M. J., Saliuk T., Scutelniciuc O., Shelley G. A., Sirinirund P., Weir S., Stroup D. F. (2010). Counting hard-to-count populations: the network scale-up method for public health. Sexually Transmitted Infections.

[CR3] Bernard HR, Johnsen EC, Killworth PD, Robinson S (1991). Estimating the size of an average personal network and of an event subpopulation: Some empirical results. Social Science Research.

[CR4] Billari FC, Giuntella O, Stella L (2018). Broadband internet, digital temptations, and sleep. Journal of Economic Behavior & Organization.

[CR5] Billari Francesco C., Giuntella Osea, Stella Luca (2019). Does broadband Internet affect fertility?. Population Studies.

[CR6] Brass W (1975). Methods for estimating fertility and mortality from limited and defective data.

[CR7] Brewer D. D., Potterat J. J., Garrett S. B., Muth S. Q., Roberts J. M., Kasprzyk D., Montano D. E., Darrow W. W. (2000). Prostitution and the sex discrepancy in reported number of sexual partners. Proceedings of the National Academy of Sciences.

[CR8] Cesare N, Lee H, McCormick T, Spiro E, Zagheni E (2018). Promises and pitfalls of using digital traces for demographic research. Demography.

[CR9] Clarke GRG, Wallsten SJ (2006). Has the internet increased trade? Developed and developing country evidence. Economic Inquiry.

[CR10] Cohen, R. A., & Adams, P. F. (2011). *Use of the internet for health information: United States, 2009* (NCHS Data Brief, No. 66). Hyattsville, MD: U.S. Department of Health and Human Services, Centers for Disease Control and Prevention, National Center for Health Statistics.

[CR11] Eckman S, Kreuter F, Kirchner A, Jäckle A, Tourangeau R, Presser S (2014). Assessing the mechanisms of misreporting to filter questions in surveys. Public Opinion Quarterly.

[CR12] Fatehkia M, Kashyap R, Weber I (2018). Using Facebook ad data to track the global digital gender gap. World Development.

[CR13] Feehan, D. M. (2015). *Network reporting methods* (Unpublished doctoral dissertation). Princeton University, Princeton, NJ. Retrieved from https://search.proquest.com/docview/1744835684

[CR14] Feehan DM, Salganik MJ (2016). Generalizing the network scale-up method: A new estimator for the size of hidden populations. Sociological Methodology.

[CR15] Feehan DM, Salganik MJ (2016). Surveybootstrap: Tools for the bootstrap with survey data.

[CR16] Feehan DM, Umubyeyi A, Mahy M, Hladik W, Salganik MJ (2016). Quantity versus quality: A survey experiment to improve the network scale-up method. American Journal of Epidemiology.

[CR17] Friemel TN (2016). The digital divide has grown old: Determinants of a digital divide among seniors. New Media & Society.

[CR18] Goel, S., Obeng, A., & Rothschild, D. (2015). *Non-representative surveys: Fast, cheap, and mostly accurate* (Working paper). Retrieved from http://adamobeng.com/download/FastCheapAccurate.pdf

[CR19] Greenwell F, Salentine S (2018). Module 8: Population-based surveys. Health information system strengthening: Standards and best practices for data sources.

[CR20] Haight M, Quan-Haase A, Corbett BA (2014). Revisiting the digital divide in Canada: The impact of demographic factors on access to the internet, level of online activity, and social networking site usage. Information, Communication & Society.

[CR21] Hill K, Trussell J (1977). Further developments in indirect mortality estimation. Population Studies.

[CR22] Hjort J, Poulsen J (2019). The arrival of fast internet and employment in Africa. American Economic Review.

[CR23] ICF. (2004). *Demographic and Health Surveys* (various) [Data sets]. Rockville: ICF [Distributor].

[CR24] ICF (2018). What we do: Survey process.

[CR25] ITU. (2018). *Percentage of individuals using the internet*. Geneva, Switzerland: International Telecommunications Union. Retrieved from https://www.itu.int/en/ITU-D/Statistics/Documents/statistics/2018/Individuals_Internet_2000-2017.xls

[CR26] Kho, K., Lakdawala, L. K., & Nakasone, E. (2018). *Impact of internet access on student learning in Peruvian schools* (Working Papers, No 2018-3). East Lansing: Michigan State University, Department of Economics.

[CR27] Lazer D., Pentland A., Adamic L., Aral S., Barabasi A.-L., Brewer D., Christakis N., Contractor N., Fowler J., Gutmann M., Jebara T., King G., Macy M., Roy D., Van Alstyne M. (2009). SOCIAL SCIENCE: Computational Social Science. Science.

[CR28] Manacorda, M., & Tesei, A. (2016). *Liberation technology: Mobile phones and political mobilization in Africa* (CESifo Working Paper Series No. 5904). Retrieved from https://papers.ssrn.com/sol3/papers.cfm?abstract_id=2795957

[CR29] Marsden, P. V. (2005). Recent developments in network measurement. In P. J. Carrington, J. Scott, & S. Wasserman (Eds.), *Models and methods in social network analysis* (pp. 8–30). Cambridge, UK: Cambridge University Press.

[CR30] Mossong Joël, Hens Niel, Jit Mark, Beutels Philippe, Auranen Kari, Mikolajczyk Rafael, Massari Marco, Salmaso Stefania, Tomba Gianpaolo Scalia, Wallinga Jacco, Heijne Janneke, Sadkowska-Todys Malgorzata, Rosinska Magdalena, Edmunds W. John (2008). Social Contacts and Mixing Patterns Relevant to the Spread of Infectious Diseases. PLoS Medicine.

[CR31] NIC.br. (2016). ICT households 2015: Survey on the use of information and communication technologies in Brazilian households.

[CR32] Ofcom. (2016). *Adults’ media use and attitudes report*. London, UK: Ofcom.

[CR33] Parsons, V. L., Moriarity, C. L., Jonas, K., Moore, T. F., Davis, K. E., & Tompkins, L. (2014). *Design and estimation for the national health interview survey, 2006–2015* (Vital and Health Statistics Report, Series 2 No. 165). Hyattsville, MD: National Center for Health Statistics. 24775908

[CR34] Perrin, A., & Duggan, M. (2015). *Americans’ internet access: 2000–2015* (Report). Washington, DC: Pew Research Center.

[CR35] Pew Research Center (2018). Internet/broadband fact sheet.

[CR36] Rao JNK, Wu CFJ (1988). Resampling inference with complex survey data. Journal of the American Statistical Association.

[CR37] Rao JNK, Wu CFJ, Yue K (1992). Some recent work on resampling methods for complex surveys. Survey Methodology.

[CR38] Rojas, G. (2015). *Harnessing technology to streamline data collection*. Rockville, MD: DHS Program, ICF. Retrieved from https://blog.dhsprogram.com/harnessing-technology-streamline-data-collection/

[CR39] Sirken MG (1970). Household surveys with multiplicity. Journal of the American Statistical Association.

[CR40] Tourangeau, R., Kreuter, F., & Eckman, S. (2015). Motivated misreporting: Shaping answers to reduce survey burden. In U. Engel (Ed.), *Survey measurements: Techniques, data quality and sources of error* (pp. 24–41). Frankfurt, Germany: Campus Verlag.

[CR41] Van Deursen AJ, Van Dijk JA (2014). The digital divide shifts to differences in usage. New Media & Society.

[CR42] Vigdor JL, Ladd HF, Martinez E (2014). Scaling the digital divide: Home computer technology and student achievement. Economic Inquiry.

[CR43] World Bank (2016). World development report 2016: Digital dividends.

[CR44] Zagheni, E., & Weber, I. (2012). You are where you e-mail: Using e-mail data to estimate international migration rates. In *Proceedings of the 4th annual ACM web science conference* (pp. 348–351). New York, NY: ACM. 10.1145/2380718.2380764

