# Supplementary material for: Using an Online Sample to Estimate the Size of an Offline Population
Source: Demography. 2019 Dec 3;56(6):2377–92. doi: 10.1007/s13524-019-00840-z (PMC6914715; doi:10.1007/s13524-019-00840-z)
Supplement: Supplementary file 1 — (PDF 994 kb) [file 13524_2019_840_MOESM1_ESM.pdf]

Online Appendix

A Derivation of the estimators

Sampling setup

We assume a conventional probability sampling setup, following the theory of design-based sampling; see Sarndal et al. (2003) for an overview. When we refer to an estimator as 'consistent', we mean design-consistent (also called Fisher consistent; Sarndal et al. (2003)). Similarly, 'unbiased' means design-unbiased.

Our frame population F – the set of people who could potentially be sampled – is monthly active Facebook users in a given country[10]. The population whose size we are trying to estimate is H, the number of internet users in the country. The goal is to use information about people on Facebook's reported offline personal network connections in order to estimate the size of H.

We assume that we obtain a *probability sample* s from the frame population, where we use the same definition of a probability sample as Sarndal et al. (2003). To briefly review, we assume that the sample s is chosen from among the members of the frame population F using a known random sampling method. The probability that $i \in F$ is included in the sample s, called i's *inclusion probability*, is written π_i. We require that $\pi_i > 0$ for all $i \in F$. We call the $w_i = \frac{1}{\pi_i}$ the *expansion weight* for unit $i \in F$.

Several of the estimators we study are ratio or compound ratio estimators. The literature on design-based sampling has established that if each component estimator is consistent and unbiased, then compound ratio estimators are design-consistent but, strictly speaking, compound ratio estimators are not unbiased. Fortunately, a large literature has studied this problem and such estimators are typically found to be very nearly unbiased, both in theory and in practice[11]. Thus, we refer to these compound ratio estimators as *essentially unbiased.* The following result formally establishes these important properties of compound ratio estimators; which we will use these properties below.

Result A.1. *Suppose that $\widehat{y}_1, \ldots, \widehat{y}_n$ are estimators that are consistent and unbiased for $Y_1, \ldots, Y_n$ respectively. Then the compound ratio estimator*

[10] Throughout this paper, we use the term Facebook users to refer to monthly-active Facebook users.

[11] We do not expect the situations in which compound ratio estimators would be biased to be relevant to our study; the biggest concern is typically when the denominator of $\widehat{R}$ is very small, which is not likely in our applications.

$$\widehat{R} = \frac{\widehat{y}_1 \cdots \widehat{y}_k}{\widehat{y}_{k+1} \cdots \widehat{y}_n}. \tag{8}$$

is consistent and essentially unbiased for $R = (Y_1 \ldots Y_k)/(Y_{k+1} \ldots Y_n)$.

Proof. See Rao and Pereira (1968), Wolter (2007) (pg. 233), and Feehan and Salganik (2016a) for more details. □

We adhere to the notation used in previous papers about network scale-up and network reporting (Feehan 2015; Feehan and Salganik 2016a; Feehan et al. 2016):

- $y_{i,B}$ is the number of reported connections from person i to members of group B
- $y_{A,B} = \sum_{i \in A} y_{i,B}$ is the number of reported connections from members of group A to group B
- $d_{i,B}$ is the number of undirected connections in the social network between i and members of group B
- $d_{A,B} = \sum_{i \in A} d_{i,B}$ is the total number of undirected connections in the social network between members of group A and members of group B
- $v_{i,A}$ is the *visibility* of i to group A – i.e., the number of times that i would be reported if everyone in A was interviewed
- $v_{B,A} = \sum_{i \in B} v_{i,A}$ is the total visibility of members of group B to group A
- $\widehat{y} \to Y$ is shorthand for '$\widehat{y}$ is a consistent and unbiased estimator for Y'
- $\widehat{y} \rightsquigarrow Y$ is shorthand for '$\widehat{y}$ is a consistent and essentially unbiased estimator for Y'
- $y^{+}_{F,H}$ is the number of reported connections from F to H that actually lead to H. If $y^{+}_{F,H} = y_{F,H}$ then we say that there are *no false positive reports*
- N_A is the size of set A (i.e., the number of people in A)

Aggregate reporting framework

We develop an estimator using the network reporting framework, an approach that builds upon insights from several different streams of previous research on sampling (Bernard et al. 1991; Feehan 2015; Feehan and Salganik 2016a; Lavallee 2007; Sirken 1970). Feehan (2015) shows that researchers can develop estimators based on network reports using either an individual or an aggregate multiplicity approach. Since we do not collect information at the level of detail required by individual multiplicity estimation, we adopt an aggregate multiplicity approach in this study. This aggregate multiplicity approach is similar to the network scale-up method (Bernard et al. 2010, 1991; Feehan and Salganik 2016a; Maltiel et al. 2015).

Result A.2. *Suppose that a census of the frame population F is interviewed and asked to report about their connections to a group Z. Call the total number of reported connections $y_{F,Z}$ and suppose $y_{F,Z} > 0$. Further, suppose that there are no false positive reports, so that $y_{F,Z} = y^{+}_{F,Z}$. Finally, suppose that $\bar{v}_{Z,F}$ is the average visibility of members of Z; that is, $\bar{v}_{Z,F}$ is the average number of times that a member of Z is reported by someone in F. Then*

$$N_H = \frac{y_{F,H}}{\bar{v}_{H,F}}. \tag{9}$$

Proof. See Feehan (2015) and Feehan and Salganik (2016a). □

To see the intuition behind the aggregate multiplicity approach from Result A.2, suppose we conducted a census of the frame population, asking every frame population member to tell us how many members of her personal network were online. Simply adding up the number of reported connections to internet users would produce a number that is larger than the number of internet users because each internet user can be reported more than once. Thus, in order to adjust for this over-counting, aggregate multiplicity estimators divide an estimate for the total number of reports by an estimate of hidden population members' *visibility.* The visibility is the number of times an average member of the hidden population would be reported if everyone on the frame population responded to the survey. In this study, the visibility is the number of times that the average internet user in a given country would be reported as an internet user, if everyone on Facebook in the country responded to the survey. Dividing the estimated total number of reported connections to people on the internet by the estimated visibility adjusts for the over-counting that would occur if the reports were used to directly estimate the number of internet users.

Given the aggregate multiplicity identity, our basic approach is to develop data collection strategies and statistical estimators that enable us to estimate the numerator and denominator of the identity in Eq. 9. In the remainder of this Appendix, we develop necessary technical results to use the identity in Eq. 9 to estimate the number of internet users in a given country.

Estimates about detailed alters

Result A.3 formalizes a situation where respondents are sampled and then asked about a sample of their network members. Result A.3 is stated in terms of an arbitrary dichotomous trait z that respondents report about their personal network members; for example, z could be Facebook usage, internet usage, gender, or membership in an age group.

Result A.3. *Suppose we have a sample s taken from the frame population using a probability sampling design. Call the expansion weights given by the sampling design w_i for each $i \in s$. Further, suppose that for each $i \in s$, we obtain information from a simple random subsample s_i of size r_i from the d_i people in i's personal network. Let z_{ij} be an indicator variable for whether or not i reports that j has trait Z, and let $z_i = \sum_{j \in s_i} z_{ij}$ be the total number of detailed alters respondent i reports having trait Z. Then the estimator*

$$\widehat{y}_{F,Z} = \sum_{i \in s} w_i \frac{d_i}{r_i} z_i \tag{10}$$

is consistent and unbiased for $y_{F,Z}$, the total number of reported connections to people with trait Z in a census of the frame population in which respondents report about everyone in their networks.

Proof. First, we note that we can consider this to be a multi-stage sample, where the first stage(s) lead to selection of the respondent and the final stage is the subsampling of detailed alters within each respondent's network. Since the final stage is a simple random sample of r_i out of d_i network members, the design weight for the final stage is $\frac{d_i}{r_i}$ for each detailed alter. In order to show that the estimator is unbiased, we take expectations with respect to the multi-stage sampling design:

$$\begin{aligned} \mathbb{E}[\widehat{y}_{F,Z}] &= \mathbb{E}_I[\sum_{i \in s} w_i \mathbb{E}_i[\frac{d_i}{r_i} z_i | s]] \\ &= \sum_{i \in F} \pi_i w_i \mathbb{E}_i[\frac{d_i}{r_i} z_i | s]] \\ &= \sum_{i \in F} \pi_i w_i \left(\sum_{j \sim i} \pi^i_j \frac{d_i}{r_i} z_{ij} \right), \end{aligned} \tag{11}$$

where the outer expectation $\mathbb{E}_I[\cdot]$ is taken with respect to the sampling of respondents and the inner expectation $\mathbb{E}_i[\cdot|s]$ is taken with respect to the sampling of detailed alters within each sampled respondent; $j \sim i$ indexes over all of the network members j that i could potentially report about; and we have written π_i for the inclusion probability of respondent i under the sampling design, and π^i_j for the inclusion probability of respondent i's jth network member under the subsampling design.

By definition, $w_i = \frac{1}{\pi_i}$ and $\pi^i_j = \frac{r_i}{d_i}$. Thus, continuing from above, we have

$$
\begin{aligned}
\mathbb{E}[\widehat{y}_{F,Z}] &= \sum_{i \in F} \pi_i w_i \left(\sum_{j \sim i} \pi^i_j \frac{d_i}{r_i} z_{ij} \right) \\
&= \sum_{i \in F} \left(\sum_{j \sim i} \pi^i_j \frac{d_i}{r_i} z_{ij} \right) \\
&= \sum_{i \in F} y_{i,Z} \\
&= y_{F,Z}.
\end{aligned} \tag{12}
$$

So we have shown that the estimator is unbiased for $y_{F,Z}$.

Finally, in a census of the frame population where every respondent reports about all of her network members, $s = F$, $\pi_i = 1$, $\pi^i_j = 1$, $z_i = y_{i,Z}$, and $r_i = d_i$ for all i and j. Thus

$$
\widehat{y}_{F,Z} = \sum_{i \in s} w_i \frac{d_i}{r_i} z_i = \sum_{i \in F} y_{i,Z} = y_{F,Z} \tag{13}
$$

So the estimator is design-consistent. □

Corollary A.1. *Under the conditions of Result A.3, the estimator*

$$
\widehat{\bar{y}}_{F,Z} = \frac{\sum_{i \in s} w_i \frac{d_i}{r_i} z_i}{\sum_{i \in s} w_i} \tag{14}
$$

is consistent and essentially unbiased for $\bar{y}_{F,Z}$.

Proof. By Result A.3, the numerator is consistent and unbiased for $y_{F,Z}$, and the denominator is a sample-based estimate for the size of the frame population, $\widehat{N}_F = \sum_{i \in s} w_i$. Thus, this is a Hajek-type estimator. See (Sarndal et al. 2003) for a proof that Hajek estimators are consistent and essentially unbiased. □

Note that Result A.3 implies that Eq. 2 is consistent and unbiased for $y_{F,H}$ and Corollary A.3 implies that Eq. 4 is consistent and unbiased for $\bar{y}_{F,F}$.

Assembling the estimator

The next estimator, Result A.4, shows that if we can estimate the total reported connections from frame population members to internet users, and if we can estimate the average visibility of internet users to frame population members, then we can estimate the number of internet users.

Result A.4. *Suppose that the* $\widehat{y}_{F,H}$ *is a consistent and unbiased estimator for* $y_{F,H}$ *and that* $\widehat{\bar{y}}_{F,F}$ *is a consistent and essentially unbiased estimator for* $\bar{y}_{F,F}$*. Further, suppose that reports are accurate in aggregate, so that* $y_{F,H} = d_{F,H}$ *and* $y_{F,F} = d_{F,F}$*. Finally, suppose that*

$$\bar{d}_{H,F} = \bar{d}_{F,F}. \tag{15}$$

Then the estimator

$$\widehat{N}_H = \frac{\widehat{y}_{F,H}}{\widehat{\bar{y}}_{F,F}} \tag{16}$$

is consistent and essentially unbiased for N_H*.*

Proof. Since $\widehat{y}_{F,H} \to y_{F,H}$ and $\widehat{\bar{y}}_{F,F} \rightsquigarrow \bar{y}_{F,F}$, Result A.1 shows that $\widehat{N}_H = \frac{\widehat{y}_{F,H}}{\widehat{\bar{y}}_{F,F}} \rightsquigarrow \frac{y_{F,H}}{\bar{y}_{F,F}}$. It remains to show that $\frac{y_{F,H}}{\bar{y}_{F,F}}$ is equal to N_H. By the condition that reports are accurate in aggregate, $y_{F,H} = d_{F,H}$ and $y_{F,F} = d_{F,F}$. Thus,

$$\frac{y_{F,H}}{\bar{y}_{F,F}} = \frac{d_{F,H}}{\bar{d}_{F,F}}. \tag{17}$$

Next, using the condition that $\bar{d}_{F,F} = \bar{d}_{H,F}$, we have

$$\frac{d_{F,H}}{\bar{d}_{F,F}} = \frac{d_{F,H}}{\bar{d}_{H,F}} = N_H \frac{d_{F,H}}{d_{H,F}} = N_H, \tag{18}$$

where the last step follows from the fact that we are assuming a symmetric type of network tie, meaning that the number of connections from F to H must be equal to the number of connections from H to F. □

Result A.4 relies upon the condition that $\bar{d}_{H,F} = \bar{d}_{F,F}$ (Eq. 15), which requires that two quantities be equal: (1) the rate at which someone who is on the internet shares a meal with someone who is on Facebook ($\bar{d}_{H,F}$); and, (2) the rate at which someone who is on Facebook shares a meal with someone who is also on Facebook ($\bar{d}_{F,F}$). This assumption could be violated if, for example, people frequently organize sharing a meal together using Facebook (without inviting other people).

To further understand the condition in Eq. 15, note that since $F \subset H$ (i.e., everyone on Facebook is also on the Internet), it follows that

$$\bar{d}_{H,F} = p_{F|H}\bar{d}_{F,F} + (1 - p_{F|H})\bar{d}_{H-F,F} \tag{19}$$

where $p_{F|H} = \frac{N_F}{N_H}$ is the prevalence of F among H, i.e., the fraction of people on the internet that is also on Facebook. Therefore, when the condition in Eq. 15 holds, then it is also the case that

$$\bar{d}_{F,F} = \bar{d}_{H-F,F}. \tag{20}$$

Appendix C introduces a sensitivity framework that researchers can use to assess how sensitive size estimates are to this condition, and Appendix F introduces simple models that motivate this condition.

B Internal consistency checks

The internal consistency checks start from an identity that relates two quantities: (1) $d_{F_{-\alpha},F_{\alpha}}$, the population-level number of connections from everyone who is in F but not group α to everyone who is in F and in group α; and (2), $d_{F_{\alpha},F_{-\alpha}}$ – the population-level number of connections from everyone who is in F and group α to everyone who is in F but not in group α. Since the networks we ask respondents to report about are symmetric, these two quantities are identical; however, they can be estimated independently from the data we collected: the first quantity can be estimated only from respondents who are not in group α, and the second quantity can be estimated only from respondents who are in group α.

In order to assess how internally consistent reporting is, we can directly compute a survey-based estimates for the discrepancy

$$\Delta^0_{\alpha} = \widehat{d}_{F_{-\alpha},F_{\alpha}} - \widehat{d}_{F_{\alpha},F_{-\alpha}}. \tag{21}$$

The closer this quantity is to 0, the more internally consistent reports about group α are. However, Δ^0_{α} is influenced by the size of the group α, which makes it challenging to plot internal consistency checks for several different groups in the same place (e.g. Fig. 4). Thus, we propose rescaling the

IC checks for different groups to put them on a more similar scale. Specifically, we scale Δ^0_α by a factor K given by

$$K = \frac{N_F}{N_{F_{-\alpha}} N_{F_\alpha}}, \tag{22}$$

where $N_{F_{-\alpha}}$ is the number of people in the frame population not in group α and N_{F_α} is the number of people in the frame population who are in group α. The factor K is motivated by starting from the identity $d_{F_{-\alpha},F_\alpha} = d_{F_\alpha,F_{-\alpha}}$, and multiplying both sides by $\frac{1}{N_{F_{-\alpha}} N_{F_\alpha}}$. The result is an expression that shows that $\bar{d}_{F_{-\alpha},F_\alpha}/N_{F_\alpha} = \bar{d}_{F_\alpha,F_{-\alpha}}/N_{F_{-\alpha}}$. In words, this new expression equates (1) the proportion of F_α that the average person in $F_{-\alpha}$ is connected to; and (2) the proportion of $F_{-\alpha}$ that the average person in F_α is connected to. Finally, we multiply the new identity by N_F to help compare countries of different sizes.

Note that this rescaling does not affect whether or not the confidence intervals for the IC checks includes 0; instead, it controls for the relative size of group α. It makes internal consistency checks across different groups easier to compare with one another.

Using the example of the conversational contact reports, the final discrepancy measure is defined to be

$$\Delta^{\text{cc}}_\alpha = K \left[\widehat{d}_{F_{-\alpha},F_\alpha} - \widehat{d}_{F_\alpha,F_{-\alpha}} \right]. \tag{23}$$

Eq. 23 can be computed for each bootstrap resample; the distribution of $\Delta^{\text{cc}}_\alpha$ across bootstrap resamples is then an estimate for the sampling distribution of the discrepancy measure.

C Sensitivity framework

In this Appendix, we describe a framework that can be used to assess the sensitivity of the estimated number of people who use the internet to the various conditions that the results in Appendix A rely upon.

In order to develop the sensitivity framework, we adapt previous work on network scale-up and other network reporting methods (Feehan 2015; Feehan and Salganik 2016a). We start by introducing three quantities, called *adjustment factors*:

$$\eta_H = \frac{\substack{\text{avg \# reported connections from} \\ \text{F to H that actually lead to H}}}{\substack{\text{avg \# reported connections} \\ \text{from F to H}}} = \frac{y^+_{F,H}}{y_{F,H}}, \tag{24}$$

and

$$\eta_F = \frac{\substack{\text{avg \# reported connections from} \\ \text{F to F that actually lead to F}}}{\substack{\text{avg \# reported connections} \\ \text{from F to F}}} = \frac{y^{+}_{F,F}}{y_{F,F}}, \tag{25}$$

and

$$\nu = \frac{\text{avg \# in-reports to H from F}}{\text{avg \# in-reports to F from F}} = \frac{\bar{v}_{H,F}}{\bar{v}_{F,F}}. \tag{26}$$

Each of these new parameters is equal to 1 under ideal conditions, when the requirements of the results in Appendix A are satisfied. In general, ν can take on any value from 0 to ∞, while η_F and η_H can take on any value from 0 to 1.

The first sensitivity result reveals how estimated numbers of internet users will be affected if one or more of the three adjustment factors is not equal to 1.

Result C.1. *Suppose that the sampling conditions for Result A.3 hold, but that the reporting and network structure conditions do not. That is, suppose we have a sample s taken from the frame population using a probability sampling design. Call the expansion weights given by the sampling design w_i for each $i \in s$. Further, suppose that for each $i \in s$, we obtain information from a simple random subsample s_i of r_i out of the d_i people in i's personal network.*

Now suppose that $\widehat{y}_{F,H}$ is consistent and unbiased for $y_{F,H}$ and that $\widehat{\bar{y}}_{F,F}$ is consistent and unbiased for $\bar{y}_{F,F}$, but that $\eta_{F,H} \neq 1$, $\eta_{F,F} \neq 1$, and $\nu \neq 1$; that is, assume that the remaining conditions in Result A.4 do not hold. Then the estimator

$$\widehat{N}_H = \frac{\widehat{y}_{F,H}}{\widehat{\bar{y}}_{F,F}} \tag{27}$$

is consistent and unbiased for $(\frac{\eta_F}{\eta_H}\nu)N_H$.

Proof. The proof follows along the lines of Feehan and Salganik (2016a). Briefly,

$$\widehat{N}_H = \frac{\widehat{y}_{F,H}}{\widehat{\bar{y}}_{F,F}} \qquad \frac{y_{F,H}}{\bar{y}_{F,F}} \tag{28}$$

by the sampling conditions. Next, we wish to use the adjustment factors to relate the estimand

to N_H:

$$\begin{aligned}
\frac{y_{F,H}}{\bar{y}_{F,F}} &= \frac{\eta_F}{\eta_H} \; \frac{y^+_{F,H}}{\bar{y}^+_{F,F}} \\
&= \frac{\eta_F}{\eta_H} \; \frac{v_{H,F}}{\bar{v}_{F,F}} \\
&= \frac{\eta_F}{\eta_H} \; \frac{\bar{v}_{H,F}}{\bar{v}_{F,F}} \; N_H \\
&= \frac{\eta_F}{\eta_H} \; \nu \; N_H.
\end{aligned} \tag{29}$$

Thus, we conclude that

$$\widehat{N}_H \rightsquigarrow \frac{\eta_F}{\eta_H} \nu N_H. \tag{30}$$

□

Corollary C.1. *Under the conditions listed in Result C.1,*

$$Bias[\widehat{N}_H] = \mathbb{E}[\widehat{N}_H] - N_H = N_H(\frac{\eta_F}{\eta_H}\nu - 1). \tag{31}$$

Now we show how problems with the sampling weights can affect estimates; this will be helpful in understanding what impact non simple random subsampling of detailed alters would have.

First, we must define *imperfect sampling weights*. We follow Feehan and Salganik (2016a) and repeat the definition here for convenience:

Imperfect sampling weights. Suppose a researcher obtains a probability sample s from the frame population F. Let I_i be the random variable that assumes the value 1 when unit $i \in F$ is included in the sample s, and 0 otherwise. Let $\pi_i = \mathbb{E}[I_i]$ be the true probability of inclusion for unit $i \in F$, and let $w_i = \frac{1}{\pi_i}$ be the corresponding design weight for unit i. We say that researchers have *imperfect sampling weights* when researchers use imperfect estimates of the inclusion probabilities π'_i and the corresponding design weights $w'_i = \frac{1}{\pi'_i}$. Note that we assume that both the true and the imperfect weights satisfy $\pi_i > 0$ and $\pi'_i > 0$ for all i.

Result C.2. *Suppose researchers have obtained a probability sample s, but that they have imperfect sampling weights. Call the imperfect sampling weights $w'_i = \frac{1}{\pi'_i}$, call the true weights*

$w_i = \frac{1}{\pi_i}$, and define $\epsilon_i = \frac{w'_i}{w_i} = \frac{\pi_i}{\pi'_i}$. Then

$$Bias[\widehat{y}'_{F,Z}] = N_F \left[\bar{y}_{F,Z}(\bar{\epsilon} - 1) + cov_F(y_{i,Z}, \epsilon_i)\right], \tag{32}$$

where $\bar{\epsilon} = \frac{1}{N_F}\sum_{i \in F} \epsilon_i$ and $cov_F(\cdot, \cdot)$ is the finite population unit covariance in the frame population F.

Proof. See Result D.2 in Feehan and Salganik (2016a). □

Result C.2 will be useful to us because we can use it to understand situations in which respondents' reports about the detailed alters are different from simple random sampling. In order to isolate the impact of such a difference, we assume in Result C.2 that the expansion weights for respondent inclusion are accurate.

We also state the following fact, which will be useful in the subsequent derivation.

Fact C.1.

$$\sum_{i \in A} a_i b_i = N_A[\bar{a}\bar{b} + \text{cov}_A(a_i, b_i)] \tag{33}$$

Result C.3. *Suppose that respondents do not report about the detailed alters by picking r_i out of d_i of them uniformly at random, so that the estimator for $\widehat{y}_{F,Z}$ in Result A.3 uses imperfect weights $l'_{ij} = \frac{d_i}{r_i}$ for the final-stage subsampling of detailed alters, while the true weight for each of respondent i's detailed alters j is given by l_{ij}. Let $\epsilon_i = \frac{l'_i}{l_i}$. Suppose also that the expansion weights w_i for the inclusion of respondents in the sample are accurate. Then the bias of $\widehat{y}'_{F,Z}$ is given by*

$$Bias[\widehat{y}'_{F,Z}] = \sum_{i \in F}\sum_{j \sim i} z_{ij}(\epsilon_{ij} - 1). \tag{34}$$

Proof.

$$\begin{aligned}
\mathbb{E}[\widehat{y}'_{F,Z}] &= \mathbb{E}\left[\sum_{i \in s} w_i \times \mathbb{E}_i[\sum_{j \in s_i} l'_{ij} z_{ij} | s]\right] \\
&= \sum_{i \in F} w_i \mathbb{E}[I_i] \times \sum_{j \sim i} \mathbb{E}_i[I_{ij}|s] l'_{ij} z_{ij} \\
&= \sum_{i \in F}\sum_{j \sim i} \frac{l'_{ij}}{l_{ij}} z_{ij} \\
&= \sum_{i \in F}\sum_{j \sim i} \epsilon_{ij} z_{ij},
\end{aligned} \tag{35}$$

where $j \sim i$ indexes the people j that are reported in respondent i's network. Thus, the bias is

$$
\begin{aligned}
\text{Bias}(\widehat{y}'_{F,Z}) &= \mathbb{E}[\widehat{y}'_{F,Z}] - y_{F,Z} \\
&= \sum_{i \in F} \sum_{j \sim i} \epsilon_{ij} z_{ij} - \sum_{i \in F} \sum_{j \sim i} z_{ij} \\
&= \sum_{i \in F} \sum_{j \sim i} z_{ij} (\epsilon_{ij} - 1).
\end{aligned}
\tag{36}
$$

□

To understand Result C.3 better, we manipulate the expression for Bias$[\widehat{y}'_{F,Z}]$ with the aim of producing a more interpretable expression:

$$
\begin{aligned}
\text{Bias}(\widehat{y}'_{F,Z}) &= \sum_{i \in F} \sum_{j \sim i} z_{ij} (\epsilon_{ij} - 1) \\
&= \sum_{i \in F} y_i \left[\bar{z}_i (\bar{\epsilon}_i - 1) + \text{cov}_{j \sim i}(z_{ij}, \epsilon_{ij} - 1) \right] \\
&= \sum_{i \in F} y_i \bar{z}_i \bar{\epsilon}_i - \sum_{i \in F} y_i \bar{z}_i + \sum_{i \in F} y_i \sigma_i \\
&= \sum_{i \in F} z_i \bar{\epsilon}_i + \sum_{i \in F} y_i \sigma_i - \sum_{i \in F} z_i,
\end{aligned}
\tag{37}
$$

where $j \sim i$ indexes the people j that are reported in respondent i's network; $y_i = y_{i,U} = \sum_{j \sim i} 1$ is the total number of people i would report about if there was no subsampling; $z_i = \sum_{i \sim j} z_{ij}$ is the total number of people i would report as members of Z if there was no subsampling; $\bar{z}_i = y_i^{-1} \sum_{i \sim j} z_{ij}$ is the average z_{ij} among respondent i's reported network members; $\bar{\epsilon}_i = y_i^{-1} \sum_{i \sim j} \epsilon_{ij}$ is the average ϵ_{ij} among respondent i's reported network members; $\bar{\epsilon} = N_F^{-1} \sum_{i \in F} \bar{\epsilon}_i$ is the average $\bar{\epsilon}_i$ across people in the frame; and $\sigma_i = \text{cov}_{i \sim j}(z_{ij}, \epsilon_{ij})$ is the covariance between the ϵ_{ij} and z_{ij} among respondent i's reported network members.

Finally, we use Fact C.1 twice–once within respondent and once between respondents:

$$
\begin{aligned}
&\sum_{i\in F} z_i\bar{\epsilon}_i + \sum_{i\in F} y_i\sigma_i - \sum_{i\in F} z_i \\
&\qquad = N_F[\bar{z}\bar{\epsilon} + \mathrm{cov}_F(z_i,\bar{\epsilon}_i)] + N_F[\bar{y}_{F,U}\bar{\sigma} + \mathrm{cov}_F(y_i,\sigma_i)] - y_{F,Z} \\
&\qquad = y_{F,Z}\left[\underbrace{(\bar{\epsilon}-1)}_{\substack{\text{aggregate}\\\text{error}\\\text{in weights}}} + \underbrace{\frac{\bar{\sigma}\ \bar{y}_{F,U} + \mathrm{cov}_F(y_i,\sigma_i)}{\bar{y}_{F,Z}}}_{\substack{\text{relationship between}\\\text{personal network}\\\text{size and}\\\text{weight errors}}} + \underbrace{\frac{\mathrm{cov}_F(z_i,\bar{\epsilon}_i)}{\bar{y}_{F,Z}}}_{\substack{\text{relationship}\\\text{between}\\\text{weight errors}\\\text{and alters'}\\\text{internet use}}}\right],
\end{aligned}
\tag{38}
$$

where $\bar{z} = N_F^{-1}\sum_{i\in F} z_i$ is the average z_i across people in the frame. Thus, Eq. 38 shows that when respondents do not choose detailed alters uniformly at random, the resulting bias can be decomposed into three terms: one term related to aggregate errors in the weights; one term that captures the relationship between personal network size and weight errors; and one term that captures the relationship between weight errors and alters' internet use.

D Additional details about survey design

In this appendix, we provide additional details about the design of our survey. We hope that these details will help researchers who wish to use our design as a starting point for future research.

Weighting and post-stratification

The estimator we develop in the main text is based on having a probability sample of the frame population. We obtained a probability sample using Facebook's internal survey sampling mechanism. However, like any real-world sampling approach, the actual set of people we interview is the result of a two-step process: first, we randomly chose people to be included in the sample; and, second, some of those people agreed to participate in the study. If the people who agreed to participate were systematically different from people who did not, that could affect the accuracy of our inferences (just like any survey). Therefore, we adjust the sampling weights using post-stratification to improve the representativeness of our sample[12]. Post-stratification is commonly used in sample surveys with the goal of helping to reduce the bias and variance of estimates; readers who are interested in learning more about post-stratification can consult

[12]Note that our goal is not to use post-stratification to account for differences between the people who use Facebook and other people; instead, the goal is to ensure that our sample is as representative as possible of people who use Facebook.

standard survey research texts (e.g., Särndal and Lundström 2005; Lumley 2011; Valliant et al. 2013). We provide a conceptual overview of how we used post-stratification here.

Our *design weights* come from the sampling design, which we treat as a simple random sample without replacement. Under this sampling design, the probability of inclusion, i.e., the probability that a given person i who uses Facebook is included in the sample, is

$$\pi_i^0 = \frac{n}{N_F},$$

where n is the sample size in i's country and N_F is the size of the frame population, i.e., the number of active Facebook users in i's country. The design weight is the reciprocal of the probability of inclusion, $w_i^0 = \frac{1}{\pi_i^0}$.

In any particular sample $s \subset F$, the set of respondents may be different from the overall population of Facebook users. To account for this fact, post-stratification adjusts these design weights by using known counts of active Facebook users by age and sex. For a given survey respondent i who is in age-sex group α, the post-stratified weight is given by

$$w_i = w_i^0 \; K_\alpha,$$

where K_α is a factor given by

$$K_\alpha = \frac{N_{F_\alpha}}{\sum_{i \in s_\alpha} w_i^0}.$$

$\sum_{i \in s_\alpha} w_i^0$ is the sum of the design weights among respondents who are in age-sex group α, and N_{F_α} is the number of people who actively use Facebook in age-sex group α (or, more generally, the size of the frame population in group α). The intuition is that the denominator is the design-weighted estimate of the number of frame population members in group α, while N_{F_α} is the actual size of group α, which is known. K_α thus adjusts the design weights so that they agree with the known total sizes.

Recall that, in our study, we estimate sampling variation using the rescaled bootstrap (Rao and Wu 1988; Rao et al. 1992). We combine post-stratification and the bootstrap by post-stratifying the set of weights that results from each bootstrap resample by age and sex[13]. Thus, after post-stratification, for each bootstrap resample the sum of survey weights will conform to known frame population totals. For example, the sum of weights among female respondents in Brazil

[13]Specifically, used the `calibrate` function in the `survey` R package (Lumley 2004, 2011).

will equal the number of females who are active on Facebook in Brazil.

In our study, post-stratifying the weights did not make a big difference in our estimates. To illustrate this fact, Figure S1 compares estimated internet adoption using the design weights to estimated internet adoption using the post-stratified weights; the estimates are very similar to one another.

However, in other studies whose goal is obtain a sample from an online frame population, we can envision situations in which post-stratification might make a bigger difference. Since we partnered with Facebook directly, we are able to obtain an actual probability sample of frame population members to invite to our survey. In general, this may not always be possible and so researchers may wish to try to recruit survey participants using incomplete lists of mobile phone numbers, online ads, or other sources from which a probability sample is not possible. In all cases, we recommend that researchers (i) critically assess the extent to which their sampling mechanism produced a representative sample of the online frame population; and (ii) consider using post-stratification or other calibration approaches to adjust systematic differences between the sample and the frame population.

Sampling designs for future studies

As we discuss above, we analyze our results as a simple random sample without replacement, and our frame population—i.e., the group of people who were eligible to be included as respondents in the survey—was people who actively use Facebook in each of the five countries we study. It was not necessary to use any stratification, oversampling, or other features of more complex sample designs for our study.

Future researchers who wish to adopt our methodology may consider using more complex sampling designs, including stratification, oversampling, and so forth. Since our results are derived using the design-based sampling framework, our estimators and variance estimation approach will generalize naturally to complex samples; a text on design-based sampling will have more details (e.g., Sarndal et al. 2003).

However, we note that modified sampling designs may require some attention to the conditions that the estimator relies upon. In some cases, it may be appropriate to modify the design used in our study to be more appropriate to the setting and the quantity of interest (see also Appendix F). As an example, if researchers wished to produce estimates of internet adoption in rural and urban areas separately, then it would make sense to (i) stratify the sample by rural and urban areas; and (ii) consider changing the survey question to focus on respondents' network members who live in the same area they do. That way, rural respondents would report about rural internet use while urban respondents reported about urban internet use. In this case, it would also be

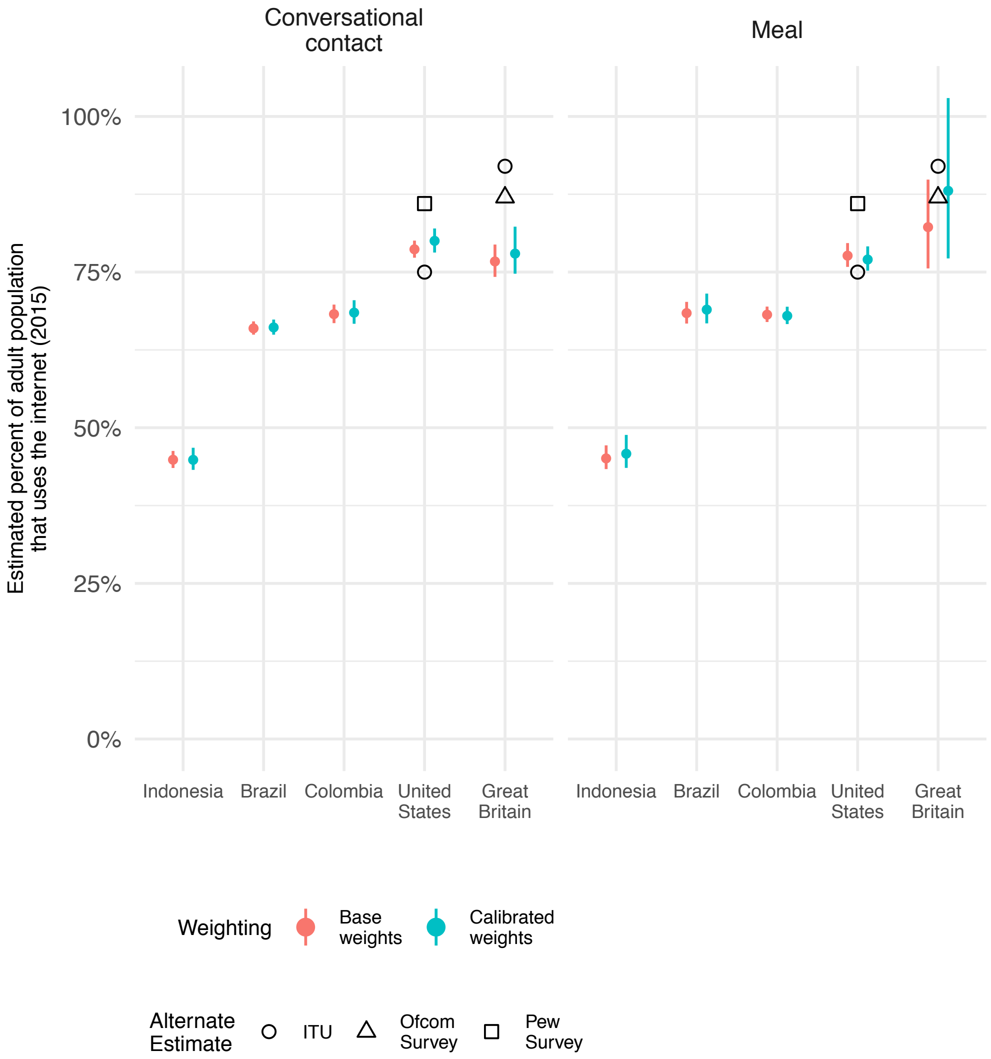

Figure S1: Estimated percentage of 2015 adult population that uses the internet, by country and for each of the two networks, with and without adjusting weights using post-stratification. The ‘base weights’ are unadjusted and the ‘calibrated weights’ are adjusted. Estimates are very similar to the ones shown in Figure 6; the main difference is that the meal estimate for Great Britain is slightly lower in this figure.

desireable for post-stratification to be conducted for age/sex/urbanicity groups, rather than just age/sex groups. Of course, other quantities of interest may require different changes to the exact design of the survey; in the same way that no single design is appropriate for all household surveys, it is also true that no single design will be appropriate for all online network reporting surveys.

Survey instrument

Figure S2 and Figure S3 show the instruments that were used in our survey. (These instruments were translated from English into the dominant local language for each country. Translated instruments were back-translated into English as a quality check, as is standard in survey research.) Nothing about the survey instrument was particularly complex, and we expect that delivering a similar survey would be possible using most existing systems.

Although we did not precisely measure the amount of time taken to complete each survey, log data suggest that the average amount of time to complete the survey was approximately 4.3 minutes for the conversational contact instrument and approximately 3.8 minutes for the meal instrument.

E Generalized estimates

Our approach relies upon survey respondents to be able to report whether or not members of their personal networks used the internet in the last 30 days. In reality, respondents may not be perfectly aware of their network members' internet use. We see two ways to address this issue. First, our sensitivity framework (Appendix C) can be used to assess how big an impact errors in reporting will have on size estimates. Second, for some groups, it may be possible to try to estimate the level of awareness of internet use from the respondents themselves (Feehan and Salganik (2016a) has an in-depth analysis of this idea applied to the network scale-up estimator). We now describe how we explored this second approach in more detail.

In our survey, we explored estimating visibility directly from the respondents by asking, for each detailed alter, whether or not the detailed alter was aware of the respondent's internet use (see the survey instrument in Figure S3). The idea is that the average extent to which detailed alters are aware of the respondents' internet use can be used to approximate awareness of internet use in general.

Mathematically, an alternate approach to estimating the number of internet users in a given country is given by

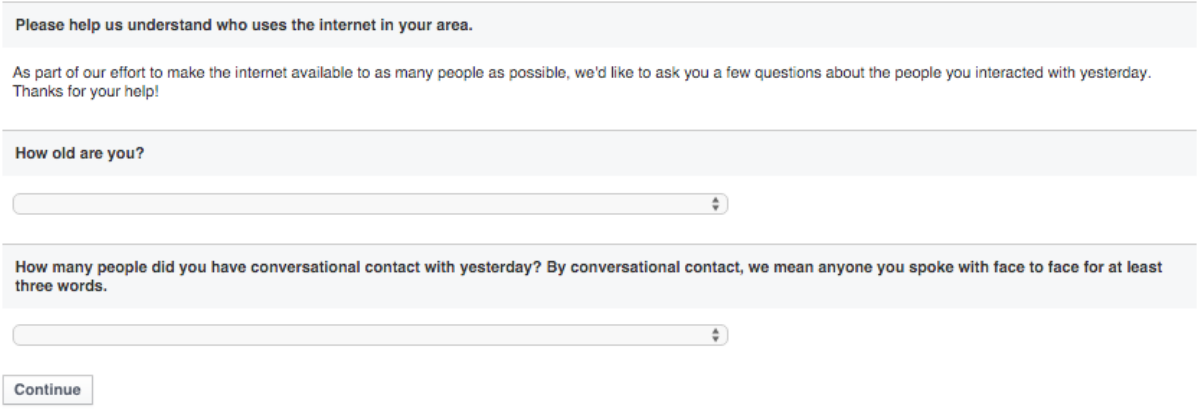
Please help us understand who uses the internet in your area.

As part of our effort to make the internet available to as many people as possible, we'd like to ask you a few questions about the people you interacted with yesterday. Thanks for your help!

How old are you?

How many people did you have conversational contact with yesterday? By conversational contact, we mean anyone you spoke with face to face for at least three words.

Continue

(a)

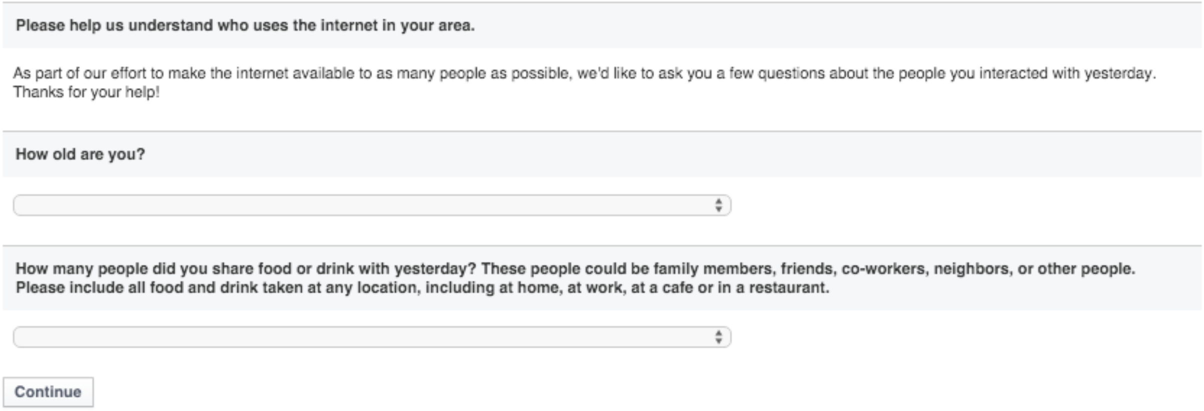
Please help us understand who uses the internet in your area.

As part of our effort to make the internet available to as many people as possible, we'd like to ask you a few questions about the people you interacted with yesterday. Thanks for your help!

How old are you?

How many people did you share food or drink with yesterday? These people could be family members, friends, co-workers, neighbors, or other people. Please include all food and drink taken at any location, including at home, at work, at a cafe or in a restaurant.

Continue

(b)

Figure S2: First page of questions shown to survey respondents in (a) the conversational contact survey; and, (b) the meal survey.

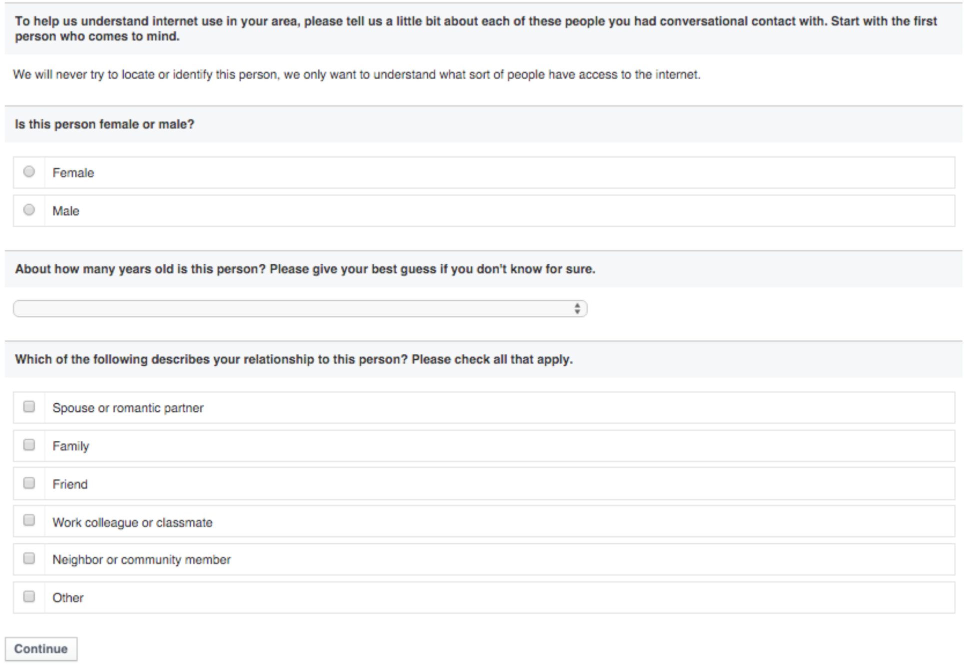
To help us understand internet use in your area, please tell us a little bit about each of these people you had conversational contact with. Start with the first person who comes to mind.

We will never try to locate or identify this person, we only want to understand what sort of people have access to the internet.

Is this person female or male?

- Female
- Male

About how many years old is this person? Please give your best guess if you don't know for sure.

Which of the following describes your relationship to this person? Please check all that apply.

- Spouse or romantic partner
- Family
- Friend
- Work colleague or classmate
- Neighbor or community member
- Other

Continue

(a)

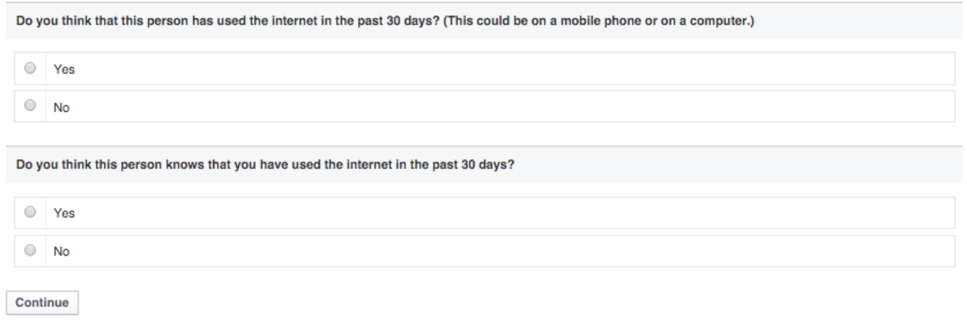
Do you think that this person has used the internet in the past 30 days? (This could be on a mobile phone or on a computer.)

- Yes
- No

Do you think this person knows that you have used the internet in the past 30 days?

- Yes
- No

Continue

(b)

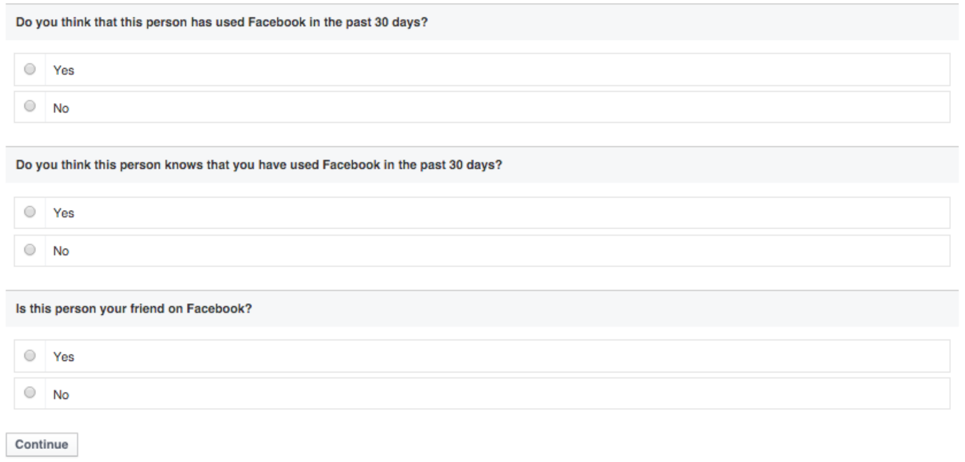
Do you think that this person has used Facebook in the past 30 days?

- Yes
- No

Do you think this person knows that you have used Facebook in the past 30 days?

- Yes
- No

Is this person your friend on Facebook?

- Yes
- No

Continue

(c)

Figure S3: Survey instrument used to ask about each detailed alter: panels (a), (b), and (c) show the first, second, and third screens asked about each of up to three detailed alters.

$$\widehat{N}_H^{\text{gen}} = \frac{\sum_{i \in s} w_i \frac{d_i}{r_i} o_i}{\sum_{i \in s} w_i \frac{d_i}{r_i} z_i}, \tag{39}$$

where the new term in the denominator, z_i, is the total number of detailed alters reported by i who are both on Facebook and reported to be aware of i's internet use. To understand Eq. 39 better, it is helpful to compare it to the estimator used in the main text (Eq. 5). The estimator in Equation 5 has in its denominator f_i, which is the number of i's detailed alters who are in the frame population. We can re-write f_i as a sum of characteristics of each detailed alter $f_i = \sum_{j \sim i} f_{ij}$, where f_{ij} is an indicator variable for whether or not i's jth detailed alter is reported to be on the frame population.

The generalized estimator in Equation 39 replaces f_i with another quantity, z_i, which is the total number of i's detailed alters who are both in the frame population and who are reported to be aware of i's own internet use. Thus, we can write $z_i = \sum_{j \sim i} f_{ij} z_{ij}$, where z_{ij} is an indicator for whether or not i's jth detailed alter is reported to be aware of i's internet use. The intuition is that z_i will be lower than f_i when many of respondents' own network members are unaware of the respondents' internet use, suggesting that general awareness about others' internet use is low. Thus, as Feehan and Salganik (2016a) explains in more detail, the main idea is to use i's perceived visibility to i's network members as a way to approximate the visibility, rather than the degree, of people on the frame population.

In practice, we found that generalized estimates were almost indistinguishable from the basic estimates with no adjustments; to illustrate this finding, Figure S4 compares the generalized estimates to the basic estimates. Thus, we focus on basic estimates in the main text. However, other study designs, tie definitions, or quantities of interest may benefit more from using a generalized estimator, and we recommend that future studies continue to explore this possibility.

F A simple model to motivate the visibility estimator

In the main text, the estimator we introduce is based upon approximating the visibility of internet users, $\bar{v}_{H,F}$ with the condition shown in Eq. 3, which we repeat here for convenience:

$$\bar{d}_{H,F} = \bar{d}_{F,F}. \tag{40}$$

Eq. 40 says that the average number of connections from an internet user to someone on Facebook equals the average number of connections from someone on Facebook to someone else on Facebook. In this Appendix, we show how a simple model can be used to derive the

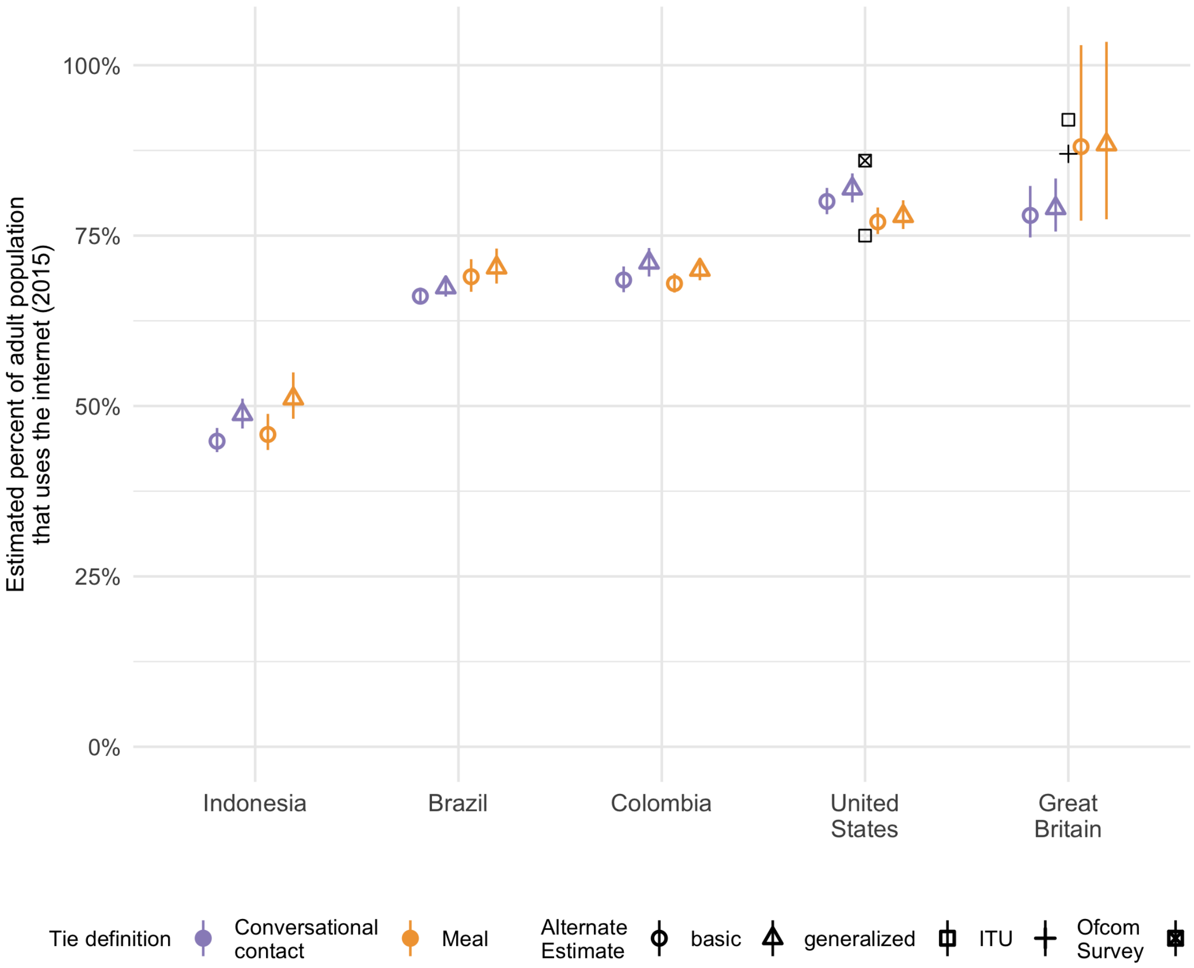

Figure S4: Comparison between basic and generalized estimates. Generalized estimates try to directly estimate visibility from the respondents, while basic estimates use the network size of survey respondents as an approximation of visibility. The figure shows that the two approaches produce very similar estimates; thus, we focus on basic estimates in the main text.

relationship in Eq. 40. We note that this model is sufficient, but not necessary: Eq. 40 is ultimately an empirical condition that can hold even if the process that generated the underlying network is quite different from this model; the goal of this model is to illustrate one simple process through which Eq. 40 would be satisfied.

For the purposes of this Appendix, we will use the meal network as our motivating example. We assume that reports are accurate, and we focus on connections among people who use the internet, i.e. members of group H (including people who use Facebook and people who don't). We ignore sampling and focus on population-level quantities, since the condition in Eq. 40 is a population-level relationship. And, in this appendix only, we will take expectations with respect to our model, and not with respect to a sampling design (unlike the rest of the paper).

The goal of the model is to make precise a situation in which people who are on the internet do not pay attention to whether or not another internet user is on Facebook when deciding to share a meal together. We call this situation *homogenous mixing*. Formally, suppose that meals are shared by each pair of internet users, $i, j \in H$ with probability p, and that the probability that any pair of internet users shares a meal is independent of everyone else's meal sharing. Then we have an Erdos-Renyi random network with parameters N_H and p. An internet user will be connected to each of the $N_F - 1$ other internet users with probability p, so the expected degree of an internet user $i \in H$ will be $\mathbb{E}[d_{i,F}] = (N_F - 1)p$.

Using the fact that $\mathbb{E}[d_{i,F}] = (N_F - 1)p$, and the independence of the edges, we can now derive expressions for both sides of the relationship in Eq. 40. Starting with $\bar{d}_{H,F}$, we find

$$
\begin{aligned}
\mathbb{E}[\bar{d}_{H,F}] &= \frac{1}{N_H}\mathbb{E}[d_{H,F}] \\
&= \frac{1}{N_H}\mathbb{E}[\sum_{i \in H} d_{i,F}] \\
&= \frac{1}{N_H}\sum_{i \in H} p(N_F - 1) \\
&= (N_F - 1)\ p.
\end{aligned} \tag{41}
$$

And, for $\bar{d}_{F,F}$, we get

$$
\begin{aligned}
\mathbb{E}[\bar{d}_{F,F}] &= \frac{1}{N_F}\mathbb{E}[d_{F,F}] \\
&= \frac{1}{N_F}\sum_{i\in F}\mathbb{E}[d_{i,F}] \\
&= \frac{1}{N_F}\sum_{i\in F}(N_F-1)p \\
&= (N_F-1)p.
\end{aligned}
\tag{42}
$$

Thus, under this model, $\bar{d}_{H,F} = \bar{d}_{F,F}$.

Extensions of the model

We will briefly discuss two ways that this simple model can be elaborated: first, we examine a situation in which the population is a mixture of different subgroups, each with different levels of internet adoption and Facebook usage; within each subgroup, homogenous mixing holds. Second, we examine a situation in which mixing is not homogenous. We see pursuing these and other elaborations of the simple model as a useful direction for future work.

Before we can extend our model, however, we must introduce two useful facts. The first fact describes how the average number of connections between two groups, A and B, can be decomposed when the first group A can be partitioned into two subgroups.

Fact F.1. Suppose that group $A \subset U$ can be partitioned into two subsets A_1 and A_2. Then for any group $B \subset U$,

$$
\bar{d}_{A,B} = p_1\bar{d}_{A_1,B} + p_2\bar{d}_{A_2,B},
\tag{43}
$$

where $p_1 = \frac{|A_1|}{|A|}$ and $p_2 = \frac{|A_2|}{|A|}$.

Fact F.1 follows from some algebra:

$$
\begin{aligned}
\bar{d}_{A,B} &= \frac{1}{|A|}\left[d_{A_1,B} + d_{A_2,B}\right] \\
&= \frac{1}{|A|}\left[|A_1|\bar{d}_{A_1,B} + |A_2|\bar{d}_{A_2,B}\right] \\
&= \frac{|A_1|}{|A|}\bar{d}_{A_1,B} + \frac{|A_2|}{|A|}\bar{d}_{A_2,B}.
\end{aligned}
\tag{44}
$$

The second fact describes how the average number of connections between two groups, A and B, can be decomposed when the second group B can be partitioned into two subgroups.

Fact F.2. Suppose that group $B \subset U$ can be partitioned into two subsets B_1 and B_2. Then for any group $A \subset U$,

$$\bar{d}_{A,B} = \bar{d}_{A,B_1} + \bar{d}_{A,B_2}. \tag{45}$$

Fact F.2 also follows from some algebra:

$$\begin{aligned} \bar{d}_{A,B} &= \frac{1}{|A|}\left[d_{A,B_1} + d_{A,B_2}\right] \\ &= \bar{d}_{A,B_1} + \bar{d}_{A,B_2}. \end{aligned} \tag{46}$$

A non-interacting mixture of different populations

Suppose the population can be partitioned into a set of groups such that (i) the number of internet users is different in each group; (ii) the proportion of internet users who use Facebook is different in each group; (iii) there are no meals shared between the groups; and, (iv) within each group, mixing is homogenous. We shall show that, in the the aggregate population, the relationship, $\bar{d}_{H,F} = \bar{d}_{F,F}$ will still hold. As a motivating example, we will consider the case in which there are rural and urban areas, and both internet adoption and Facebook usage are higher in urban areas.

Formally, call the number of Facebook users in urban and rural areas $N_{F \cap C}$ and $N_{F \cap R}$; call the number of internet users in urban and rural areas $N_{H \cap C}$ and $N_{H \cap R}$; and let the fraction of internet users that is on Facebook be $p_C = \frac{N_{F \cap C}}{N_{H \cap C}}$ for urban areas and $p_R = \frac{N_{F \cap R}}{N_{H \cap R}}$ for rural areas. Suppose that people only share meals with others who are in the same area as them; so, there are no meals shared between rural and urban areas. Finally, suppose that within these areas, homogenous mixing holds so that the condition in Eq. 40 is satisfied; that is, suppose that $\bar{d}_{H \cap R, F \cap R} = \bar{d}_{F \cap R, F \cap R}$ and $\bar{d}_{H \cap C, F \cap C} = \bar{d}_{F \cap C, F \cap C}$, where $F \cap C$ and $F \cap R$ are urban and rural Facebook users and $H \cap C$ and $H \cap R$ are urban and rural internet users.

The setup is a mixture of two populations, one urban and one rural. Within each population, the simple model studied above describes the networks. We wish to show that in the the aggregate

population, the relationship, $\bar{d}_{H,F} = \bar{d}_{F,F}$ holds.

Applying Fact F.1 to $\bar{d}_{H,F}$, we have

$$\begin{aligned} \bar{d}_{H,F} &= p_C \bar{d}_{H \cap C,F} + p_R \bar{d}_{H \cap R,F} \\ &= p_C \bar{d}_{H \cap C,F \cap C} + p_R \bar{d}_{H \cap R,F \cap R}. \end{aligned} \tag{47}$$

Where the last step follows from Fact F.2 together with the assumption that there are no meals shared between rural and urban areas. Next, we apply the fact that mixing is homogenous within each area, i.e., that $\bar{d}_{H \cap R,F \cap R} = \bar{d}_{F \cap R,F \cap R}$ and $\bar{d}_{H \cap C,F \cap C} = \bar{d}_{F \cap C,F \cap C}$. Eq. 47 can thus be simplified to

$$p_C \bar{d}_{H \cap C,F \cap C} + p_R \bar{d}_{H \cap R,F \cap R} = p_C \bar{d}_{F \cap C,F \cap C} + p_R \bar{d}_{F \cap R,F \cap R} \tag{48}$$

Finally, note that Fact F.2, together with the assumption that there are no meals shared between rural and urban areas, suggests substituting $\bar{d}_{F \cap R,F \cap R} = \bar{d}_{F \cap R,F}$ and $\bar{d}_{F \cap U,F \cap U} = \bar{d}_{F \cap U,F}$ into Eq. 47. This substitution produces

$$\begin{aligned} p_C \bar{d}_{F \cap C,F \cap C} + p_R \bar{d}_{F \cap R,F \cap R} &= p_C \bar{d}_{F \cap C,F} + p_R \bar{d}_{F \cap R,F} \\ &= \bar{d}_{F,F}. \end{aligned} \tag{49}$$

Thus, we have shown that $\bar{d}_{H,F} = \bar{d}_{F,F}$ in this mixture model.

The analysis above emphasizes the fact that, in this simple model, the key condition is that $\bar{d}_{H,F} = \bar{d}_{F,F}$. If Facebook and internet adoption rates are different in different types of places, but within a given place, Facebook users are evenly distributed through the network, the condition in Eq. 40 may still be a reasonable basis for approximating visibility.

Non-uniform mixing

In this section, we consider a second extension of the simple model: we generalize the model to a situation in which people who use Facebook may interact differently with people who do and who do not use Facebook. Thus, we no longer assume that mixing is homogenous. We will see that in this case, the condition $\bar{d}_{H,F} = \bar{d}_{F,F}$ will no longer hold in general.

In order to incorporate non-homogenous mixing between internet users who are and are not on Facebook, we use a block model (see, e.g., Wasserman and Faust 1994). The block model says that the probability that $i, j \in H$ are connected is a function only of the 'block' or group

$$
\begin{array}{c c} & \begin{array}{cc} F & H-F \end{array} \\ \begin{array}{c} F \\ H-F \end{array} & \begin{pmatrix} \phi & \sigma \cdot \phi \\ \sigma \cdot \phi & \phi \end{pmatrix} \end{array} \tag{50}
$$

Figure S5: Matrix describing the block model: entry (i, j) shows the probability that a randomly chosen node from group i is connected to a randomly chosen node from group j. The matrix is parameterized by $\phi \in [0, 1]$, the probability of a connection between two members of the same group; and $\sigma \in [0, 1]$, the extent to which the probability of connectivity is diminished when two nodes are in different groups. When $\sigma = 1$, we have the homogenous mixing model considered above; when $\sigma < 1$, there is non-homogenous mixing.

memberships of i and j. We consider a block model with two groups: Facebook users (F) and internet users who do not use Facebook ($H - F$). Every internet user is in one and only one of these two groups. Figure S5 shows the probability that i is connected to j given the group memberships of i (rows) and j (columns) in terms of two parameters: $\phi \in [0, 1]$ and $\sigma \in [0, 1]$. The parameter ϕ controls the probability of edges between two members of the same group, and the parameter σ is a factor by which the probability of a connection is reduced between two nodes who are in different groups. When $\sigma = 1$, this block model reduces to the simple homogenous mixing model we considered above. On the other hand, when $\sigma < 1$, there is non-homogenous mixing: someone on Facebook is more likely to share a meal with someone else on Facebook than someone who is not on Facebook.

Now we will derive expressions for $\bar{d}_{F,F}$ and $\bar{d}_{H,F}$ under this block model. First, note that each node in F has a probability ϕ of being connected to each of the $N_F - 1$ other nodes in F; thus, $\mathbb{E}[\bar{d}_{F,F}] = (N_F - 1)\phi$. Also, note that if N_F is much bigger than 1, which it will typically be in the situations we are interested in, then $\mathbb{E}[\bar{d}_{F,F}] \approx N_F\phi$.

Next, we consider $\bar{d}_{H,F}$. By Fact F.1, this quantity can be written as

$$
\begin{aligned}
\mathbb{E}[\bar{d}_{H,F}] &= \mathbb{E}[p_{F|H}\ \bar{d}_{F,F} + (1 - p_{F|H})\bar{d}_{H-F,F}] \qquad \text{(by Fact F.1)} \\
&= p_{F|H}\ \mathbb{E}[\bar{d}_{F,F}] + (1 - p_{F|H})\mathbb{E}[\bar{d}_{H-F,F}],
\end{aligned} \tag{51}
$$

where we have written the proportion of internet users who also use Facebook as $p_{F|H} = \frac{N_F}{N_H}$.

Now, each node in $H - F$ will have a probability $\phi\sigma$ of being connected to each of the N_F nodes in F; thus, $\mathbb{E}[\bar{d}_{H-F,F}] = N_F\phi\sigma$. Substituting this relationship into Eq. 51, along with the approximation $\mathbb{E}[\bar{d}_{F,F}] \approx N_F\phi$ discussed above, we obtain

$$
\begin{aligned}
\mathbb{E}[\bar{d}_{H,F}] &= p_{F|H}\ \mathbb{E}[\bar{d}_{F,F}] + (1 - p_{F|H})\mathbb{E}[\bar{d}_{H-F,F}] \\
&\approx p_{F|H}\ N_F\phi + (1 - p_{F|H})N_F\phi\sigma.
\end{aligned}
\tag{52}
$$

Comparing Eq. 52 to $\bar{d}_{F,F} \approx N_F\phi$, we can see that the two will not, in general be the same; indeed, as long as $p_{F|H} < 1$ or $\sigma < 1$, they will be different.

To better understand this result, we can incorporate this analysis into our sensitivity framework. Under perfect reporting, the parameter $\eta = \frac{\bar{v}_{H,F}}{\bar{v}_{F,F}}$ can be written as $\eta = \frac{\bar{d}_{H,F}}{\bar{d}_{F,F}}$. Now we can substitute the expressions we just derived to see what the value of η would be under this block model:

$$
\begin{aligned}
\eta &= \frac{\bar{v}_{H,F}}{\bar{v}_{F,F}} \\
&= \frac{\bar{d}_{H,F}}{\bar{d}_{F,F}} && \text{(under perfect reporting)} \\
&= \frac{p_{F|H}\ (N_F - 1)\phi + (1 - p_{F|H})N_F\phi\sigma}{(N_F - 1)\phi} \\
&\approx \frac{p_{F|H}\ N_F\phi + (1 - p_{F|H})N_F\phi\sigma}{N_F\phi} && \text{(since } N_F - 1 \approx N_F\text{)} \\
&= 1 + p_{F|H}(1 - \sigma).
\end{aligned}
\tag{53}
$$

Eq. 53 shows that, under this block model, non-homogenous mixing will change the parameter η so that it is different from one. η will be farther from 1 when σ is farther from 1–that is, when there is more non-homogenous mixing–and when the share of internet users that is on Facebook, $p_{F|H}$, is smaller.

Summary

To recap, in this appendix, we introduced three simple models to help understand the condition $\bar{d}_{F,F} = \bar{d}_{H,F}$, which is the basis of our approach to approximating the visibility of internet users in the main text. First, we saw that under a model in which people on the internet mix homogenously, paying no attention to whether or not they are on Facebook, this relationship would be expected to hold. Next, we saw that if the population is a mixture of different populations – say, urban and rural people – then the condition can also hold, as long as the different populations don't mix, and mixing is homogenous within each population. Finally, we introduced a third model in which there is non-homogenous mixing. We saw that this third model could lead to values of the η parameter that are different from 1, meaning that the condition is violated.

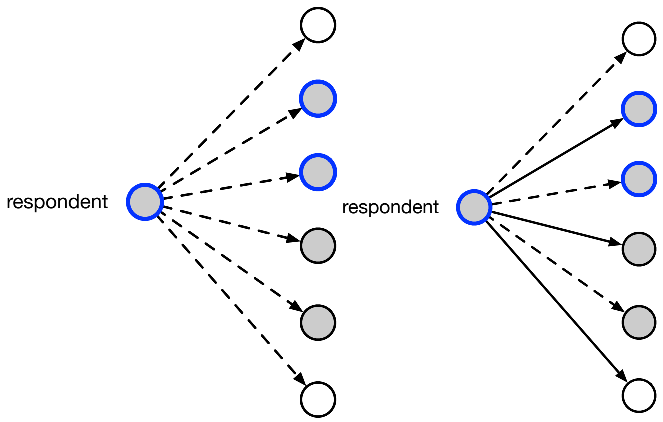

(a) (b)

Figure S6: (a) A survey respondent who is sampled online can be asked to report about members of one of her offline personal networks (e.g. her kin, friendship, or contact networks). Her responses contain information about both people who are online and people who are offline. (b) In order to reduce respondent burden, we asked for more detailed information about internet use, gender, and age for three *detailed alters* in each respondent's personal network (solid lines).

G Additional results

Table S2 reports estimated average network size (degree) for each tie definition.

country	Conversational contact	Meal
Brazil	13.1 (12.5, 13.6)	6.3 (5.9, 6.6)
Colombia	10.5 (10, 11.1)	7.2 (6.9, 7.6)
Great Britain	12.7 (11.6, 13.9)	4.4 (3.7, 5.3)
Indonesia	11 (10.4, 11.6)	7.5 (7, 8)
United State	12.1 (11.6, 12.5)	5 (4.6, 5.4)

Table S2: Estimated average degree and 95% confidence interval, by type of personal network

Fig. S6 illustrates the detailed alters subsampled from each respondent's personal network.

Table S3 provides a summary of the comparison of TAE between the two tie definitions within each country (the information that is visualized in Fig. 5).

Country	Median TAE	Mean TAE (95% CI)
Brazil	8.62	8.64 (2.16, 15.54)
Colombia	4.77	4.82 (-1.36, 11.14)
Great Britain	5.43	5.67 (-2.58, 14.22)
Indonesia	-6.71	-6.85 (-24.13, 8.82)
United States	1.39	1.4 (-3.28, 6.52)

Table S3: Estimated mean and 95% CI for the TAE, the difference in total absolute error in internal consistency checks across all age groups for the conversational contact network minus the same quantity for the meal network. Positive values mean that the conversational contact network was less internally consistent than the meal network, as measured by absolute error.

References

Bernard, H. R., Hallett, T., Iovita, A., Johnsen, E. C., Lyerla, R., McCarty, C., . . . Stroup, D. F. (2010). Counting hard-to-count populations: The network scale-up method for public health. *Sexually Transmitted Infections, 86*(Suppl. 2), ii11–ii15. http://sti.bmj.com/content/86/Suppl_2/ii11.short

Bernard, H. R., Johnsen, E. C., Killworth, P. D., & Robinson, S. (1991). Estimating the size of an average personal network and of an event subpopulation: Some empirical results. *Social Science Research, 20,* 109–121.

Feehan, D. M. (2015). *Network reporting methods* (Unpublished doctoral dissertation). Princeton University, Princeton, NJ. Retrieved from https://search.proquest.com/docview/1744835684

Feehan, D. M., & Salganik, M. J. (2016a). Generalizing the network scale-up method: A new estimator for the size of hidden populations. *Sociological Methodology, 46,* 153–186.

Feehan, D. M., Umubyeyi, A., Mahy, M., Hladik, W., & Salganik, M. J. (2016). Quantity versus quality: A survey experiment to improve the network scale-up method. *American Journal of Epidemiology, 183,* 747–757.

Lavallee, P. (2007). *Indirect sampling*. New York, NY: Springer-Verlag.

Lumley, T. (2004). Analysis of complex survey samples. *Journal of Statistical Software, 9*(1), 1–19.

Lumley, T. (2011). *Complex surveys: A guide to analysis using R*. Hoboken, NJ: John Wiley & Sons.

Maltiel, R., Raftery, A. E., McCormick, T. H., & Baraff, A. J. (2015). Estimating population size using the network scale up method. *Annals of Applied Statistics, 9,* 1247–1277.

Rao, J. N. K., & Pereira, N. P. (1968). On double ratio estimators. *Sankhyā: The Indian Journal of Statistics, Series A (1961–2002), 30*(1), 83–90.

Särndal, C.-E., & Lundström, S. (2005). *Estimation in surveys with nonresponse*. New York, NY: John Wiley & Sons.

Särndal, C.-E., Swensson, B., & Wretman, J. (2003). *Model assisted survey sampling*. New York, NY: Springer Verlag.

Sirken, M. G. (1970). Household surveys with multiplicity. *Journal of the American Statistical Association, 65,* 257–266.

Valliant, R., Dever, J. A., & Kreuter, F. (2013). *Practical tools for designing and weighting survey samples*. New York, NY: Springer.

Wasserman, S., & Faust, K. (1994). *Social network analysis: Methods and applications*. Cambridge, UK: Cambridge University Press.

Wolter, K. (2007). *Introduction to variance estimation* (2nd ed.). New York, NY: Springer.
